# Structural and functional constraints on spike activation and host protease utilization limit cell entry of SARS-CoV-2-related bat coronaviruses

**DOI:** 10.1128/jvi.01007-25

**Published:** 2025-07-24

**Authors:** Qingqing Li, Xiao Cai, Xiaoning Li, Yibing Zhang, Ru Li, Zirui Kang, Didi Wan, Jiaxu Wang, Lili Li, Junxia Yang, Jianxiang Shi, Shuiling Jin, Xiangdong Sun, Ying Peng, Na Zang, Zhengkun Xie, Yushun Wan, Jian Shang

**Affiliations:** 1 Henan Institute of Medical and Pharmaceutical Sciences, Zhengzhou University 12636, Zhengzhou, Henan, China; 2 College of Basic Medicine, Chongqing Medical University 12550, Chongqing, China; 3 The First Affiliated Hospital of Zhengzhou University, Zhengzhou University 12636, Zhengzhou, Henan, China; 4 College of Life Sciences, Henan Normal University 66519, Xinxiang, Henan, China; 5 The Fifth Affiliated Hospital of Zhengzhou University, Zhengzhou University 12636, Zhengzhou, Henan, China; 6 Department of Respirationtory Children’s Hospital of Chongqging Medical University, National Clinical Research Center for Child Health and Disorders, Ministry of Education Key Laboratory of Child Development and Disorders, Chongqing Key Laboratory of Child Rare Diseases in Infection and Immunity 653893, Chongqing, China; 7 College of Chemistry, Zhengzhou University 12636, Zhengzhou, Henan, China; 8 State Key Laboratory of Metabolic Dysregulation & Prevention and Treatment of Esophageal Cancer, Zhengzhou University 12636, Zhengzhou, Henan, China; St. Jude Children's Research Hospital, Memphis, Tennessee, USA

**Keywords:** cross-species transmission, membrane fusion, cryo-EM structure, receptor binding, spike, SARS-CoV-2-related coronavirus

## Abstract

The persistent threat posed by SARS-CoV-2-related coronaviruses (SC2r-CoVs) in wildlife highlights the risk of zoonotic transmission. Cross-species infectivity is predominantly determined by spike (S) character and S-mediated cell entry. In this study, we systematically investigated BANAL-52 and BANAL-103, which exhibit the closest genetic proximity to SARS-CoV-2, focusing on their spike structures and functional characteristics. First, despite comparable receptor-binding domain (RBD)-ACE2 interactions, the spikes of BANAL-52 and BANAL-103 displayed significantly reduced ACE2 binding compared to SARS-CoV-2, suggesting impaired S activation. Second, Cryo-EM structural analyses revealed that BANAL-52 S is stabilized in a “locked” state through linoleic acid (LA) binding and an additional N370 glycan, whereas BANAL-103 S adopts a “closed” conformation due to a unique glycan network. Site-directed mutagenesis targeting the LA binding pocket confirmed that Y365 is related to S conformational transitions and viral entry. Third, both BANAL spikes relied predominantly on lysosomal proteases (e.g., cathepsins) for membrane fusion, unlike SARS-CoV-2, which utilizes a broader range of proteases (e.g., TMPRSS2 and furin). The introduction of a furin cleavage site enhanced the fusogenicity of BANAL spikes. Finally, sera from individuals who have recovered from SARS-CoV-2 effectively neutralized BANAL pseudoviruses, underscoring conserved antigenicity. Our findings elucidate structural and proteolytic barriers that restrict the zoonotic potential of these viruses and propose targeted surveillance strategies to preempt the emergence of SC2r-CoVs.

**IMPORTANCE:**

The viral entry mechanisms, which are primarily related to the spike character, play a critical role in determining zoonotic potential. Among the currently identified SC2r-CoVs, BANAL-52 and BANAL-103 exhibit spike proteins with the highest sequence similarity to SARS-CoV-2, rendering them optimal models for comparative studies on S-mediated cell entry and cross-species transmission. In this study, we systematically investigated the molecular constraints governing the functionality of BANAL spikes, with a focus on S-ACE2 interactions, S activation, S structures, and host protease utilization. Notably, we resolved the cryo-EM structure of BANAL-52 S at neutral pH and the first cryo-EM structure of BANAL-103 S, revealing distinct glycan- and lipid-mediated stabilization of inactive states. Furthermore, cross-neutralization assays demonstrated that sera of convalescents from SARS-CoV-2 inhibited BANAL pseudovirus entry with an efficiency of approximately 80%, thereby highlighting conserved antigenic epitopes and informing the development of broad-spectrum therapeutic strategies against emerging SC2r-CoVs.

## INTRODUCTION

The emergence and spread of coronaviruses pose a significant threat to the health of both humans and animals. Over the past two decades, three major coronavirus outbreaks that significantly impacted human health were all associated with cross-species transmission (1). The SARS-CoV outbreak in 2002–2003 originated in bats and was transmitted to humans via civets, resulting in over 8,000 infections with a case fatality rate of approximately 10% (2, 3). The MERS-CoV outbreak in 2012, which caused more than 2,000 infections and a case fatality rate of 35%, is believed to have originated in bats and been transmitted to humans through camels (4, 5). The emergence of SARS-CoV-2 at the end of 2019 has caused unprecedented disruption to global public health and economic development (6), also likely representing a cross-species transmission event, though its exact transmission pathway and intermediate hosts remain unidentified.

Epidemiological evidence indicates the widespread presence of coronaviruses closely related to SARS-CoV, MERS-CoV, and SARS-CoV-2 in wildlife reservoirs, particularly bats. While these viruses currently exhibit limited capacity for sustained human transmission, this limitation is attributed to molecular barriers such as suboptimal spike protein architecture (7), reduced receptor-binding affinity (8), incomplete adaptation to host proteases (9), and challenges in evading human immune defenses (10). However, the continuous accumulation of spontaneous viral mutations, combined with increasing human encroachment into wildlife habitats, substantially elevates the risk of zoonotic spillover events. As a result, animal-origin coronaviruses continue to pose a persistent threat to global health, emphasizing the critical need for proactive surveillance and intervention strategies.

The cell entry of coronavirus into host cells is a critical determinant of their infectivity and cross-species transmission, which is mainly mediated by the conformational change of the spike protein involving receptor recognition and membrane fusion (11). Conformational changes in the coronavirus spike receptor-binding domain (RBD) are closely associated with receptor binding efficiency and immune evasion. A previous study reported the “locked” and “closed” states of the SARS-CoV-2 spike protein, characterized by the RBD-down conformation (12). The primary distinguishing features between these two states include the presence of lipid-accessory (LA) molecules bound within the lipid-binding pocket of the RBD and an ordered fusion peptide proximal region (FPPR) in the “locked” state (13). However, we observed variability in the structures of previously reported “closed” states, necessitating further classification. Some structures exhibit tighter trimeric packing (14), resembling the “locked” state except for the absence of LA binding (15); these were retained under the designation of “closed state S.” In contrast, other structures display looser trimeric packing (16), similar to the packing trace in “open state S” (RBD-up conformation) (17), and were classified as “activated state S.”

Receptor recognition occurs through sequential conformational changes in the spike protein: transitioning from a locked conformation to a closed conformation, then to an activated conformation, followed by an open conformation, and finally binding to the cellular receptor (15). Membrane fusion is initiated by the cleavage of the spike through host proteases, leading to conformational changes in the S2 subunit (18). Each step of receptor recognition and membrane fusion significantly influences viral infectivity and cross-species transmission.

Successful cross-species transmission often results from host-adaptive mutations in the spike protein. Some mutations facilitate spike transition to an activated state. For example, the D614G mutation enhances the open conformation of SARS-CoV-2 spike and alters viral fitness (19–21). Some make spike RBD obtain higher RBD-receptor binding affinity, such as the N501Y mutation, which increases RBD-receptor binding affinity, expanding the host range of SARS-CoV-2 (22, 23). Some enhance adaptation to host proteases, such as furin (24–27), TMPRSS2 (28), and lysosomal proteases (29). Despite these insights, the precise rules or mechanisms governing coronavirus cross-species transmission remain elusive due to the lack of a systematic study. A global investigation into the characteristics of zoonotic coronaviruses, particularly focusing on the spike and spike-mediated cell entry, could provide valuable insights into predicting host adaptive mutation and cross-species transmission events.

Here, we comparatively investigated the characteristics of the spike structure, receptor binding, membrane fusion, host protease adaptation, and cross-antigenicity of two SC2r-CoVs, BANAL-52 and BANAL-103. This revealed the key restriction for BANAL spike activation and S-mediated cell entry and provided insights for viral cross-species transmission.

## RESULTS

### Characteristics of receptor binding by viral RBDs and spikes

Coronaviruses isolated from *Rhinolophus pusillus* bats in northern Laos have been reported to exhibit the closest genetic relationship with SARS-CoV-2 (30). Among these, BANAL-52 and BANAL-103 display the highest sequence similarity in spike RBD to SARS-CoV-2 (97.67% and 97.16%, respectively) (Fig. S1A). Focusing on the S receptor-binding motifs (RBMs), there is only one amino acid difference (Q498H) between the SARS-CoV-2 and BANAL spikes (Fig. S1B to S1D), which has been reported to increase the affinity of the SARS-CoV-2 RBD for hACE2 and be involved in host range expansion (30).

**Fig 1:**
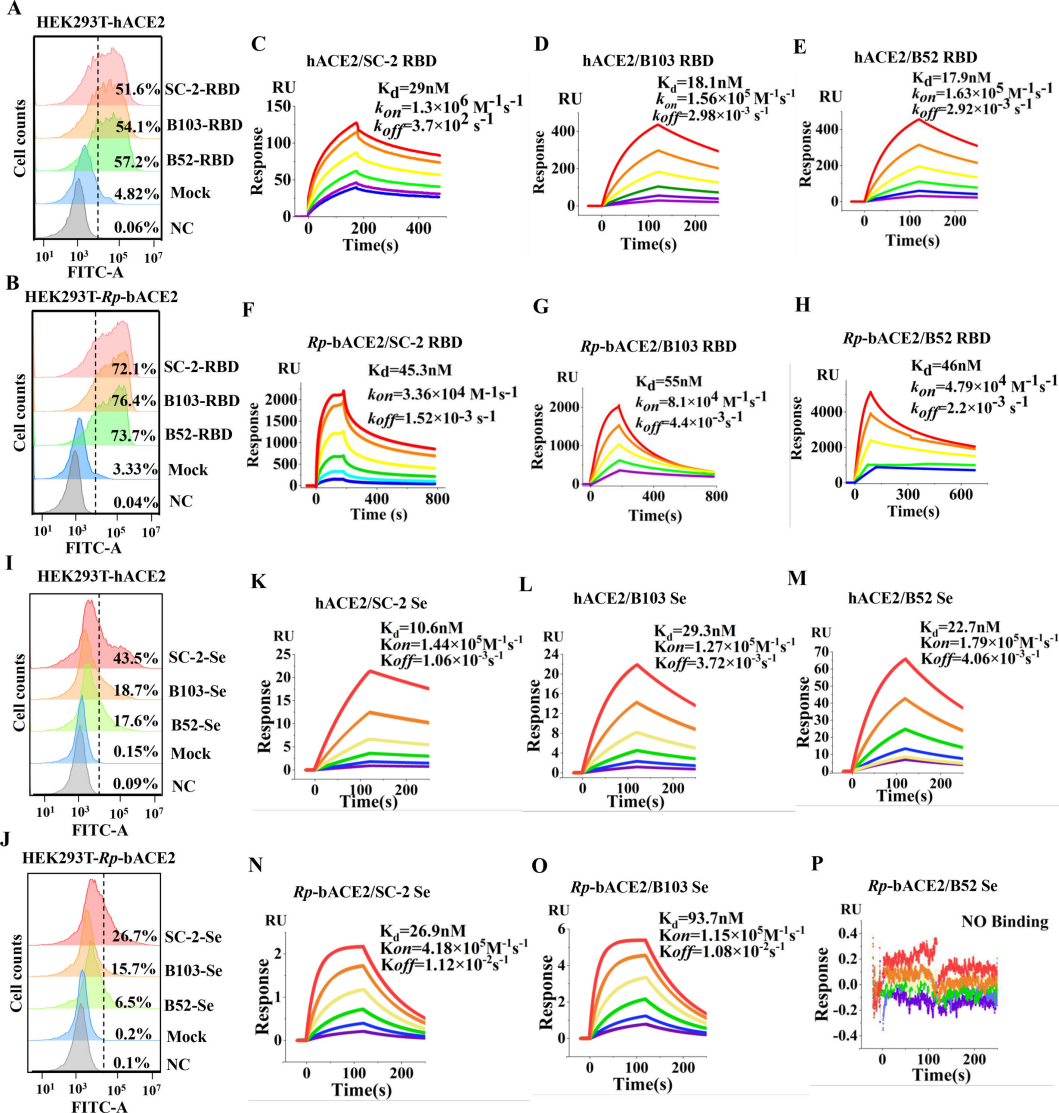
Receptor binding characteristics of BANAL S-RBDs and spike proteins. (**A and B**) Flow cytometry analysis of the binding of SARS-CoV-2 (SC-2), BANAL-52 (B52), and BANAL-103 (B103) RBD to cell surface-expressing hACE2 or Rp-bACE2. (**C–E K–M**) SPR assay to evaluate the binding affinity of purified recombinant hACE2 to the RBDs of SARS-CoV-2 (**C**), BANAL-52 (**D**), and BANAL-103 (**E**), as well as to the spikes of SARS-CoV-2 (**K**), BANAL-52 (**L**), and BANAL-103 (**M**). (**I and J**) Flow cytometry analysis of the binding of SC-2, B52, and B103 spike ectodomain (Se) to cell surface-expressing hACE2 or Rp-bACE2. (**F–H AND N–P**) SPR assay to assess the binding affinity of purified recombinant Rp-bACE2 to the RBDs of SARS-CoV-2 (**F**), BANAL-52 (**G**), and BANAL-103 (**H**), and also to the spikes of SARS-CoV-2 (**N**), BANAL-52 (**O**), and BANAL-103 (**P**).

To explore the affinity of three viral RBDs to ACE2 derived from humans or bats, HEK293T cells were respectively overexpressed with human ACE2 (hACE2) or *Rhinolophus pusillus* bat ACE2 (Rp-bACE2). Cells were harvested and fixed 48 hours post-transfection, and three purified viral RBD recombinant proteins were applied to bind the cell surface receptors, followed by analysis via flow cytometry. The results indicated that the RBDs of BANAL-52 and BANAL-103 displayed a slight increase in hACE2 binding compared to SARS-CoV-2 (Fig. 1A), but exhibited equivalent Rp-bACE2 binding for all three RBDs (Fig. 1B). Furthermore, a surface plasmon resonance assay was conducted to analyze the precise RBD-receptor binding affinities for SARS-CoV-2, BANAL-52, and BANAL-103. The findings were consistent with the results of the flow cytometry analysis, demonstrating that Q498H had a slight binding increase in hACE2 binding (Fig. 1C through 1E), but no impact on Rp-bACE2 binding (Fig. 1F through 1H), and all the RBDs exhibited stronger binding to hACE2 than to Rp-bACE2 (Fig. 1C through 1H). It also indicated that the RBDs of BANAL-52 and BANAL-103 displayed even or higher ACE2 binding compared to the RBD of SARS-CoV-2.

Our previous research revealed that the RBD adopting an open conformation determines the trimeric S-receptor binding (31). Consequently, the binding of SARS-CoV-2, BANAL-52, and BANAL-103 spikes to hACE2 or Rp-bACE2 was separately analyzed through flow cytometry using HEK293T cells expressing the respective receptor. The results showed that the binding of SARS-CoV-2 S to hACE2 or Rp-bACE2 was significantly above that of BANAL-52 and BANAL-103 S, and the binding of BANAL-52 S to the receptor was the lowest, especially to Rp-bACE2 (Fig. 1I and 1J), indicating that SARS-CoV-2 S had more open conformations than BANAL-52 and BANAL-103 S when binding with receptors, as well as BANAL-52 S was more restricted to be activated than BANAL-103 S.

SPR assays were subsequently performed to analyze the precise spike-receptor binding differences among SARS-CoV-2, BANAL-52, and BANAL-103. The results aligned with those of the flow cytometry analysis, demonstrating that the SARS-CoV-2 spike exhibited significantly higher binding affinity compared to BANAL-52 and BANAL-103, both for hACE2 (Fig. 1K through 1M) and Rp-bACE2 (Fig. 1N through 1P). Notably, the BANAL-52 spike showed extremely weak or negligible binding to Rp-bACE2 (Fig. 1P). Therefore, BANAL spikes are more restricted in an inactivated conformation than the SARS-CoV-2 spike, particularly the BANAL-52 spike.

To further investigate the influence of receptor differences on BANAL-52 S activation, we conducted a comparative analysis of hACE2 and Rp-bACE2 at both the sequence and protein structure levels. Three amino acids (Q24K, K31D, and M82N) were identified as potentially critical for spike interaction (Fig. S1A and S1C). Subsequently, reciprocal single-point mutations were introduced into hACE2 and Rp-bACE2, resulting in six mutant constructs. All the mutant recombinant proteins were successfully expressed and purified for subsequent pull-down assays. The results indicated that the K24Q and N82M mutations on Rp-bACE2 did not enhance the binding toward BANAL-52 S, whereas the D31K mutation on Rp-bACE2 significantly increased its binding (Fig. S2D). In contrast, the Q24K and M82N on hACE2 had no discernible effect on BANAL-52 S binding, while the K31D mutation on hACE2 markedly reduced it (Fig. S2E). Furthermore, recombinant hACE2-K31D and Rp-bACE2-D31K proteins were expressed in 239 F cells and purified for SPR assays. The results are consistent with that of the pull-down assay, confirming that the D31K mutation in Rp-bACE2 significantly enhances the binding of BANAL-52 spike, increasing it from no detectable interaction (Fig. 1O) to high-affinity binding (Fig. S2H). This mutation also improves the binding of the BANAL-103 spike (Fig. S2I compared with Fig. 1P) but does not enhance the binding of the SARS-CoV-2 spike (Fig. SF). Conversely, the K31D mutation in hACE2 drastically reduces the binding affinity of the BANAL-52 spike (Fig. S2K) and moderately decreases the binding of both BANAL-103 and SARS-CoV-2 spikes (Fig. S2J and S2L). These findings suggest that residue K31 on ACE2 plays a pivotal role in promoting or stabilizing the open conformation of BANAL and SARS-CoV-2 spikes, especially the BANAL-52 spike. Consequently, BANAL-52 may more readily infect species with ACE2 variants containing K31, highlighting a key determinant in its cross-species transmission.

### Structure features of BANAL-52 and BANAL-103 spikes

**Fig 2:**
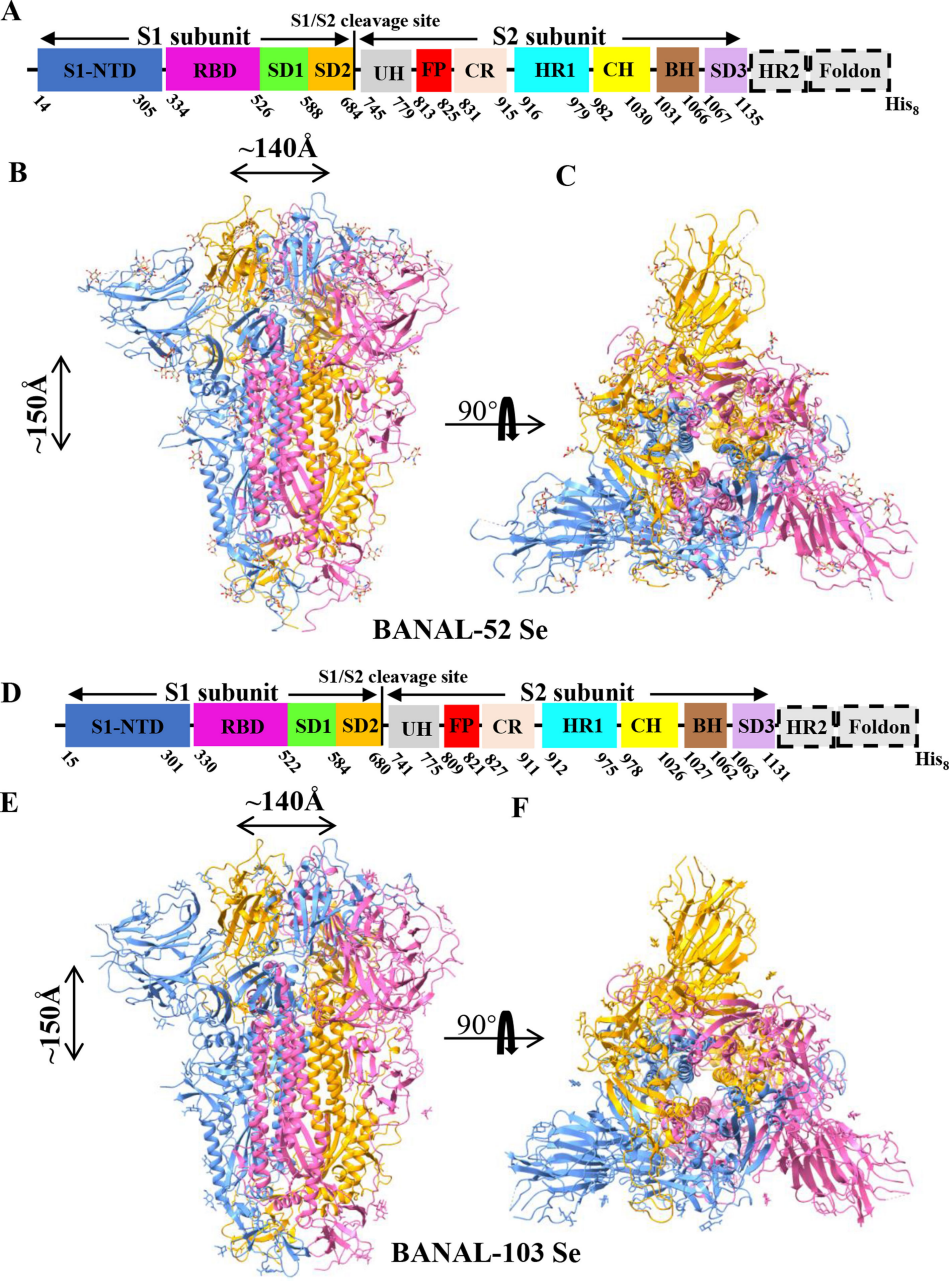
Cryo-EM structure of BANAL Spikes. (**A and D**) Schematic drawing of BANAL-52 and BANAL-103 Se. S1 subunit: receptor-binding subunit. S2 subunit: membrane-fusion subunit. Foldon His_8_: foldon trimerization tag followed by His_8_ tag. S1-NTD: N-terminal domain of S1. RBD: receptor-binding domain. SD1: subdomain 1 of S1. SD2: subdomain 2 of S1. CH: central helix. FP: fusion peptide. HR-1 and HR-2: heptad repeats 1 and 2, respectively. (**B and C**) Structure of BANAL-52 S shown in side and top views, respectively. (**E and F**) The structure of BANAL-103 S is shown in side and top views, respectively.

To directly investigate the conformation status of S proteins under physiological conditions, we employed a structural biology approach. Specifically, we engineered the BANAL-52 and BANAL-103 S ectodomains by replacing their transmembrane anchor and intracellular domain with a C-terminal foldon trimerization tag followed by a His_8_ tag (Fig. 2A and D). These proteins were expressed in 293 F cells and purified to homogeneity. Cryo-EM data were collected for both BANAL-52 and BANAL-103 S ectodomains, yielding density maps at a resolution of 3.51 Å for BANAL-52 S and 3.11 Å for BANAL-103 S (Fig. S3). Atomic models of the structures were subsequently built and refined. The final structural model for BANAL-52 S encompasses all residues from 14 to 1,140 (excluding residues 71–74, 183, 251–253, 625–630, 679–683, and 825–829) as well as glycans N-linked to 18 sites (Fig. 2B and C). For BANAL-103 S, the final model includes all residues from 15 to 1131 (excluding residues 72–74, 145–151, 246–252, and 673–680) along with glycans N-linked to 20 sites (Fig. 2E and F). Data collection and model statistics are summarized in Table S1.

**Fig 3:**
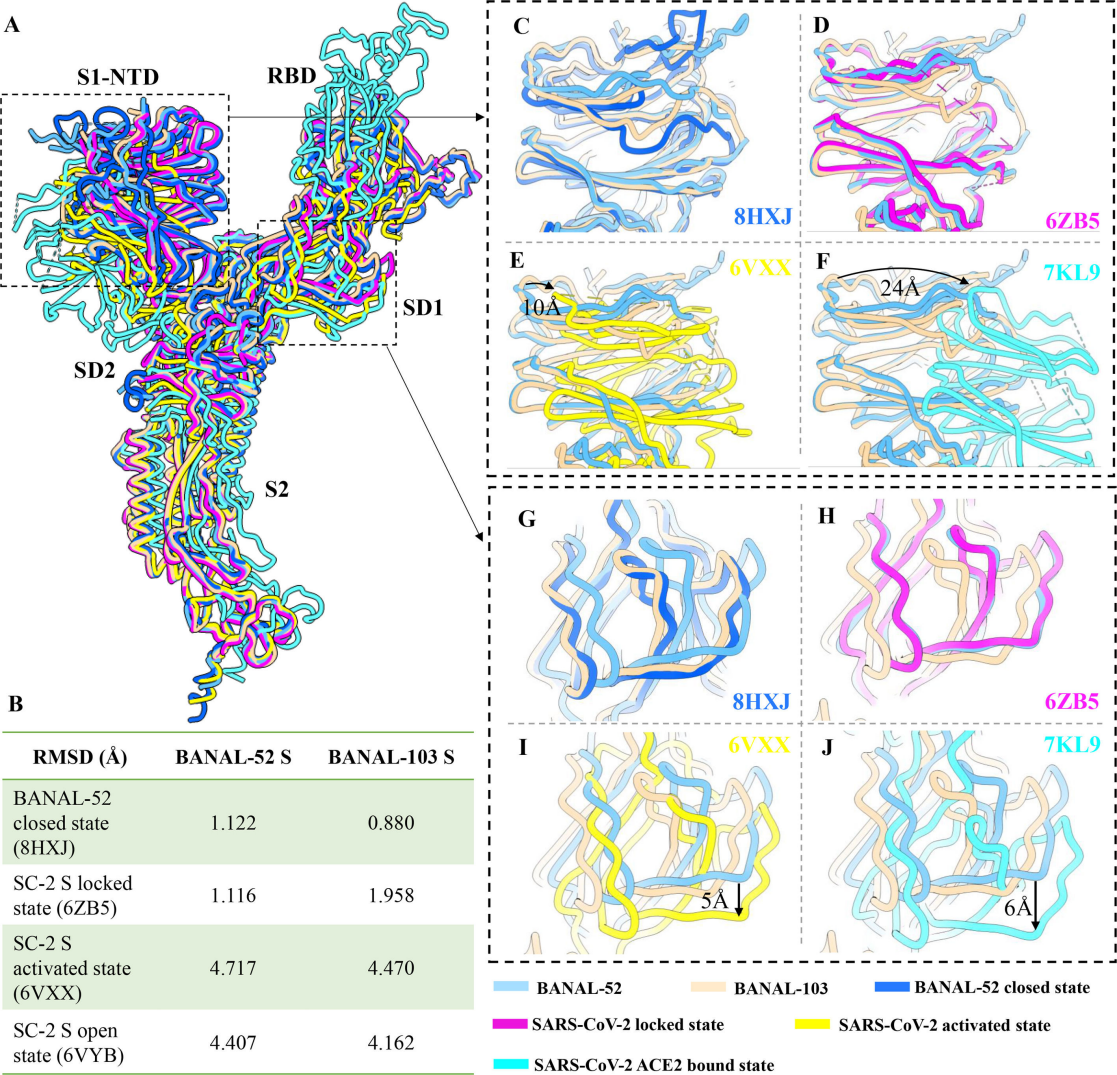
The prefusion spike conformations of BANAL-52 and BANAL-103. (**A**) Spike structure alignment of BANAL-52, BANAL-103, the previously reported closed conformation of BANAL-52 (PDB: 8HXJ), the locked conformation of SARS-CoV-2 (PDB: 6ZB5), the activated conformation of SARS-CoV-2 (PDB: 6 VXX), and the ACE2-bound conformation of SARS-CoV-2 (PDB: 7KL9). (**B**) Root-mean-square deviation (RMSD) calculated using UCSF Chimera for BANAL S structures compared to the closed conformation spike of BANAL-52 and the locked, activated, and open conformation spikes of SARS-CoV-2. (**C–F**) Close-up view of the S1-NTD region in the structural alignment. (**G–J**) Close-up view of the SD1 region in the structural alignment. Cornflower blue represents BANAL-52 S, light orange represents BANAL-103 S, heavy blue represents the closed conformation of BANAL-52 S, magenta represents the locked conformation of SARS-CoV-2 S, yellow represents the activated conformation of SARS-CoV-2 S, and cyan represents the ACE2-bound conformation of SARS-CoV-2.

The overall structure of BANAL spike ectodomains closely resembles the prefusion structures of previously reported SARS-CoV-2 and SC2-rCoV S ectodomains. Given that coronavirus prefusion S proteins exist in multiple conformations (locked, closed, activated, and open), we performed structural alignments of BANAL S proteins with the SARS-CoV-2 S in locked conformation (6ZB5), activated conformation (6 VXX), receptor-bound conformation (7KL9), and a previously reported BANAL-52 S in closed conformation (8HXJ) (Fig. 3A). The entire structure of BANAL-52 S aligns well with the SARS-CoV-2 locked conformation S, with an RMSD of 1.116 Å (Fig. 3B, D and H). Similarly, BANAL-103 S aligns well with the previously reported BANAL-52 closed-conformation S, with an RMSD of 0.88 Å (Fig. 3B, C and G). In contrast, both exhibit more compact S1 conformation compared to the SARS-CoV-2 S in activated conformation (Fig. 3E and I) and ACE2-bound conformation (Fig. 3F and J). These suggest that BANAL S proteins are likely to be in an inactivated conformation under physiological conditions, which accounts for their lower receptor binding compared to SARS-CoV-2.

### LA ligand and extra N-glycosylation restricted BANAL S conformation

**Fig 4:**
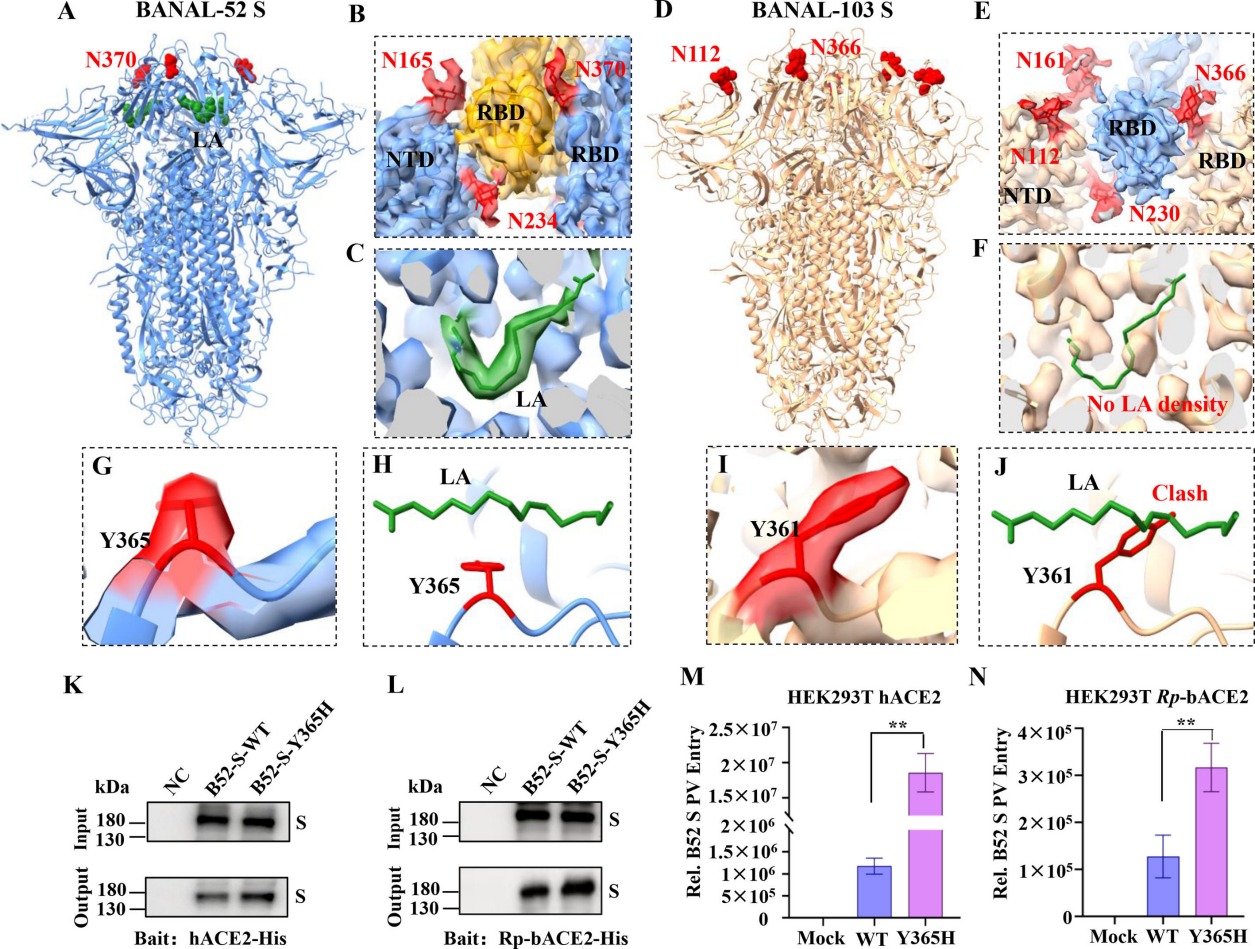
Linoleic acid (LA) binding and additional N-glycosylation in BANAL spikes. (**A**) LA and the extra N370 glycosylation site visualized in BANAL-52 S. (**B**) Triangle N-glycan pocket consisting of N-165, N234, and N370 in BANAL-52 S. (**C**) LA density shown in the BANAL-52 S cryo-EM map. (**D**) The additional N112 and N366 glycosylation sites displayed in BANAL-103 S. (**E**) Trapezoidal N-glycan pocket formed by N112, N161, N230, and N366 in BANAL-103 S. (**F**) No LA density observed in the BANAL-103 S map. (**G and H**) LA and Y365 side chain positions in the BANAL-52 S structure and map. (**I and J**) Putative LA clashed with Y361 in the BANAL-103 S structure. (**K and L**) The binding of hACE2 (**K**) or Rp-bACE2 (**L**) toward BANAL-52 S-WT and BANAL-52 S-Y365H was analyzed by pull-down assay. (**M and N**) Pseudovirus entry of BANAL-52 S-WT and BANAL-52 S-Y365H in hACE2-expressing 293T cells (**M**) or Rp-bACE2-expressing 293T cells (**N**).

Furthermore, we analyzed the N-glycosylation features of BANAL-52 and BANAL-103 in the context of their S protein structures. The S protein of BANAL-52 contains an additional N370 glycosylation site compared to SARS-CoV-2 S, which, together with N165 and N234 glycosylation sites, forms a triangular glycan pocket that stabilizes the conformation of RBD (Fig. 4A and B). Additionally, BANAL-52 S has one linoleic acid (LA) bound to a hydrophobic pocket within each RBD (Fig. 4A and C), which locks the RBD conformational changes. In contrast, for BANAL-103 S, no LA density was detected in the cryo-EM map (Fig. 4F). However, it possesses two additional N-glycosylation sites (N112 and N366) located in the NTD and RBD, respectively. These sites, along with N161 and N230, form a more compact trapezoid glycan pocket that restricts the RBD conformational changes (Fig. 4D and E). Correlating these findings with their receptor-binding capabilities, we speculate that the extra N-glycosylation sites forming a compact glycan pocket maintain BANAL-103 S in a closed conformation, whereas BANAL-52 S is stabilized in a locked conformation due to the additional binding of LA to the RBD, resulting in even lower S-receptor binding.

To validate our proposal based on structural insights, we exploited the differential sensitivity of the spike protein to protease digestion (18). A proteolysis assay was applied to SARS-CoV-2, BANAL-52, and BANAL-103 S-pseudotyped viruses to evaluate the receptor-induced S conformational changes. The results demonstrated that SARS-CoV-2 spikes were almost entirely cleaved during the pseudovirus packaging process due to the presence of a furin cleavage site (Fig. S4A, lane #1). Furthermore, the SARS-CoV-2 spikes are predominantly cleaved at the S2' site upon trypsin treatment, and this cleavage is enhanced in the presence of hACE2 (Fig. S4A, lanes #2 and #3). This indicates that the SARS-CoV-2 spike undergoes conformational changes, exposing the S2' site even in the absence of hACE2, while hACE2 further amplifies this process. For BANAL-52 and BANAL-103, their spikes can be readily cleaved by trypsin at the S1/S2 boundary (Fig. S4B and S4C, lane #2). Additionally, the cleaved spikes exhibit further proteolysis in the presence of hACE2, although no clear band at the S2' site is observed (Fig. S4B and S4C, lane #3). This suggests that both BANAL-52 and BANAL-103 likely possess trypsin-like protease cleavage sites near the S1/S2 boundary, and hACE2 induces further conformational changes. Notably, the S2 region of BANAL-103 is more extensively cleaved compared to BANAL-52 in the presence of hACE2, with a slightly higher level of S2' expression (Fig. S4C lane #3 compared to S4B lane #3). This suggests that BANAL-52 undergoes conformational changes with greater difficulty compared to BANAL-103. This is consistent with the conformational differences observed for BANAL-52 and BANAL-103 spikes.

As LA binding restricts S activation, receptor recognition, and viral infectivity (15), we compared the structural differences in the LA-binding hydrophobic pocket with and without LA bound. However, we identified only one amino acid side chain conformational difference: Y365 in BANAL-52 S (Fig. 4G) and its equivalent, Y361, in BANAL-103 S (Fig. 4I). Based on the structural analysis of BANAL S proteins, Y365 in rotamer I would accommodate LA binding (Fig. 4; Fig. S5A), while Y365/361 in rotamer II would cause a significant clash with LA (Figure 4; Fig. S5B and S5C). The same Y365 side chain conformational differences were also observed in the locked, activated, and ACE2-bound conformations of SARS-CoV-2 S structures (Fig. S5D to S5F), suggesting Y365 side chain conformation might be related to the transition from the S locked conformation to the closed conformation.

To further confirm the importance of Y365 in LA binding and S conformational change, a Y365H mutation was introduced into BANAL-52 S to disrupt the hydrophobic LA binding pocket. Without LA binding, BANAL-52 S should transition more easily from a locked conformation, enhancing viral cell entry. This was demonstrated through a pull-down assay and a pseudovirus entry assay. There was a marked increase in hACE2 or Rp-bACE2 binding for BANAL-52 S bearing the Y365H mutation (Fig. 4K and L), and the Y365H mutant S-packaged pseudovirus exhibited efficient cell entry compared to wild-type BANAL-52 S in both hACE2- and Rp-bACE2-expressing HEK293T cells (Fig. 4M and N). In conclusion, Y365 plays a critical role in LA binding, and its side chain conformation may be related to the S conformational transition, potentially serving as a barrier for viral cell entry and cross-species transmission.

A recent study has verified that sialic acid binding facilitates coronavirus S activation for receptor binding (32), and SARS-CoV-2 S1-NTD has been reported to bind sialic acid (33). We then investigated whether glycan binding was another factor triggering BANAL S proteins' conformational change. We tested bovine mucin binding by SARS-CoV-2, BANAL-52, and BANAL-103 S1-NTDs using SPR and dot blot assays. The results indicated that SARS-CoV-2 and BANAL-103 S-NTD proteins can weakly bind to bovine mucin, while BANAL-52 exhibited no apparent binding (Fig. S6). These findings suggest that BANAL-103 S, with glycan binding capacity, would be more easily activated compared to BANAL-52 S, consistent with the biochemical assay results.

### The relatively narrow spectrum of host protease utilization restricts BANAL S-mediated membrane fusion

**Fig 5:**
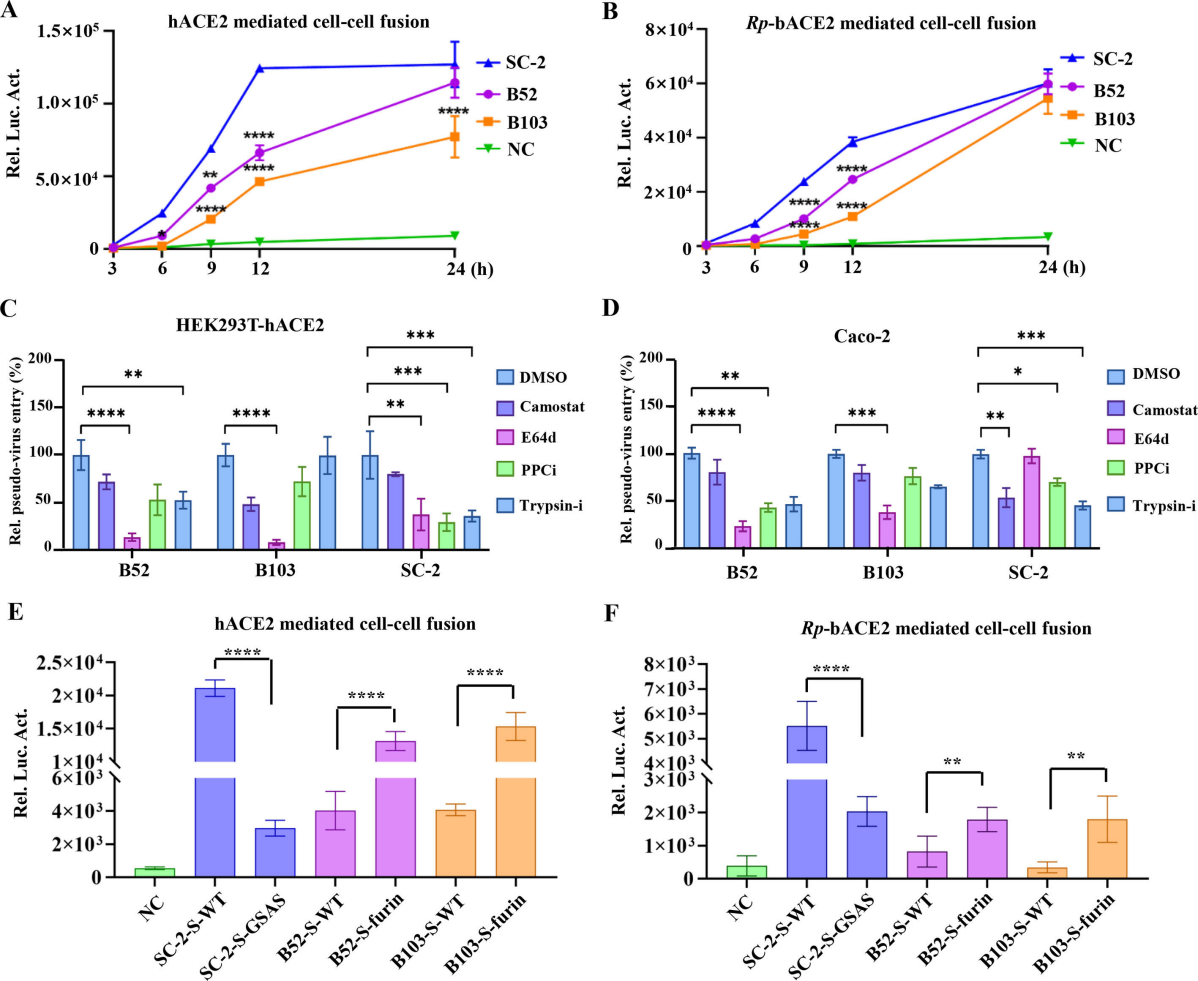
BANAL spikes mediated membrane fusion and host protease utilization. (**A and B**) SARS-CoV-2, BANAL-52, and BANAL-103 S proteins induce cell-cell fusion in HEK293T cells expressing hACE2 or Rp-bACE2. (**C and D**) Pseudovirus entry mediated by SARS-CoV-2, BANAL-52, and BANAL-103 S proteins is analyzed following treatment with protease inhibitors in hACE2-expressing HEK293T cells or Caco-2 cells. The concentration of inhibitors used is 30 µM. DMSO is used as the negative control, Camostat is used as a TMPRSS2 inhibitor, E64d used as a lysosomal cathepsin inhibitor, PPCi used as a proprotein convertase inhibitor, and trypsin-i used as trypsin-like inhibitor. (**E and F**) Cell-cell fusion induced by the SARS-CoV-2 S with a furin site mutation, as well as the BANAL-52 and BANAL-103 S with an introduced furin cleavage site, respectively, is examined in HEK293T cells expressing hACE2 or Rp-bACE2. *P < 0.05, **P < 0.01, and ***P < 0.001..

After comprehending the structural features and receptor recognition of BANAL spikes, a cell-cell fusion assay was utilized to quantify the membrane fusion capability mediated by BANAL spikes, an essential process in coronavirus cell entry. In hACE2-expressing HEK293T cells, SARS-CoV-2 S demonstrated significant cell-cell fusion within 6 h, reaching saturation at 12 h. By contrast, neither BANAL-52 nor BANAL-103 S exhibited notable cell-cell fusion until 6 h using the same assay; however, BANAL-52 S displayed stronger membrane fusion efficiency compared to BANAL-103 S (Fig. 5A). For Rp-bACE2-expressing HEK293T cells, the same trend was observed, except that membrane fusion saturation occurred later at 24 h (Fig. 5B). Nevertheless, we consistently observed a significantly lower level of S-mediated cell-cell fusion for bat viral spikes. These findings suggest that even though these spikes can bind to their corresponding receptors efficiently, they may be less effective at triggering membrane fusion compared to SARS-CoV-2 S. We speculate that a major difference in host protease utilization among SARS-CoV-2, BANAL-52, and BANAL-103 S could be the cause.

To confirm this hypothesis, protease inhibitors were applied in a pseudovirus entry assay to identify the proteases critical for S-mediated viral entry, as previously described (31). The results indicated that in hACE2-expressing HEK293T cells, all tested inhibitors significantly restricted SARS-CoV-2 S-mediated viral entry, suggesting SARS-CoV-2 can utilize a wide variety of human proteases to facilitate its entry. In contrast, only E64d treatment inhibited BANAL-103 S-mediated viral entry, indicating that BANAL-103 enters cells exclusively through the endocytosis pathway and utilizes a very narrow spectrum of host proteases in 293T cells. For BANAL-52, E64d treatment provided the most significant inhibition, while trypsin inhibitor and PPC inhibitor still had some effect, suggesting that BANAL-52 is better adapted to utilize more human proteases than BANAL-103 in 293T cells (Fig. 5C). This result was corroborated in Caco-2 cells (Fig. 5D). Previous studies reported that BANAL-52 and BANAL-236 spikes primarily depend on cell membrane TMPRSS2 and rarely utilize lysosomal proteases during entry in Calu3 (34) and HBECs-ALL (35) cells. This differs from what we observed in Caco-2 cells. To further validate our findings in Caco-2 cells, we examined the dose-dependent effects of inhibitors on the cell entry of SARS-CoV-2, BANAL-52, and BANA-103 pseudoviruses. The results demonstrated that camostat and E64d concentrations above 50 µM significantly inhibited the entry of BANAL-52 and BANAL-103 pseudoviruses in Caco-2 cells. At lower inhibitor concentrations, BANAL-52 appeared more sensitive to camostat, whereas BANAL-103 exhibited greater sensitivity to E64d (Fig. S7). Collectively, these findings indicate that SARS-CoV-2 S is well-adapted to utilize a wide range of host proteases, whereas both BANAL-52 and BANAL-103 S-mediated membrane fusion are limited primarily due to the relatively narrow spectrum of host proteases utilized.

A key step in coronavirus evolution is believed to be the acquisition of a critical protease cleavage site in its spike protein (9). We retrospectively evaluated whether such a prospective adaptation in these two SC2r-CoVs would lead to a significant increase in infectivity. A SARS-CoV-2 equivalent furin cleavage site was then introduced to BANAL-52 and BANAL-103 S. Gaining such a furin cleavage site distinctly enhanced BANAL-52 and BANAL-103 S-mediated cell-cell fusion, while the absence of a furin cleavage site reduced SARS-CoV-2 S-mediated cell-cell fusion in hACE2-expressing HEK293T cells (Fig. 5E), as well as in Rp-bACE2-expressing HEK293T cells (Fig. 5F). Collectively, these results suggest that broadening host protease utilization would be a critical requirement for enabling human cell infectivity and cross-species transmission of BANAL-52 and BANAL-103.

### Cross-neutralization of BANAL spikes packaged pseudo-viruses

Although we have a clear understanding of the restrictions on BANAL S proteins’ activation and cell entry, we cannot ignore their ongoing host-adaptive evolution aimed at breaking through these barriers. Previous studies have shown that over 90 percent of SARS-CoV-2 neutralizing antibodies are associated with its S RBD (36). Given the high similarity between BANAL S RBDs and the SARS-CoV-2 RBD, it is reasonable to assume that BANAL S RBDs exhibit cross-antigenicity with most SARS-CoV-2 S-related neutralizing antibodies.

**Fig 6:**
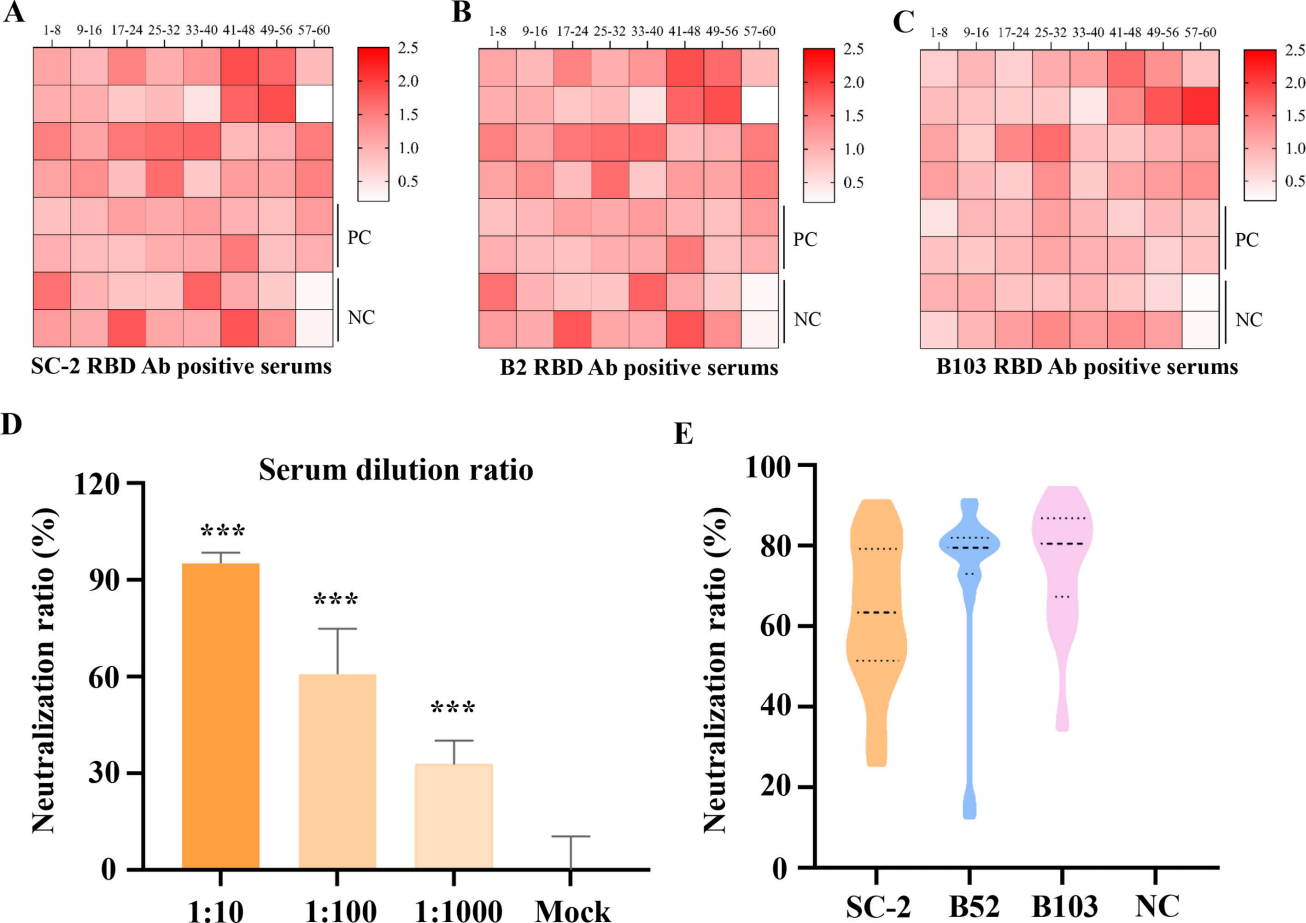
Cross-neutralization of SARS-CoV-2 S antibody-positive serum against BANALs. (**A**) Heat map showing the binding of SARS-CoV-2 RBD to 60 randomly selected serum samples. (**B and C**) Heat maps showing binding of BANAL S RBDs to the same 60 randomly selected serum samples. (**D**) Screened serum dilution ratio for the neutralization assay. (**E**) Neutralization assay of the positively selected serum (58 out of 60 samples) against SARS-CoV-2, BANAL-52, and BANAL-103 S-mediated pseudovirus cell entry. ***P < 0.001.

To validate this hypothesis, we collected plasma samples from health check-ups conducted at the Children’s Hospital of Chongqing Medical University. Using ELISA, we evaluated the presence of SARS-CoV-2 RBD-related antibodies in these samples, regardless of whether the subjects had been vaccinated or infected. The heatmap revealed that 58 out of 60 randomly selected sera exhibited positive binding to the SARS-CoV-2 RBD (Fig. 6A), indicating that over 95% of the population still possesses SARS-CoV-2 S-related antibodies. Subsequently, we tested the cross-antigenicity of BANAL S proteins against these positive sera. The results demonstrated significant cross-antigenicity for BANAL S RBDs (Fig. 6B and C), suggesting the potential for cross-neutralization.

Next, we screened an appropriate serum dilution ratio for the neutralization assay, ultimately selecting a 1:100 dilution for further testing (Fig. 6D). Pseudoviruses mediated by BANAL spikes were then utilized for the cross-neutralization assay. The selected positive sera achieved 65% neutralization for SARS-CoV-2 S-packaged pseudoviruses, while reaching approximately 80% for BANALs (Fig. 6E). This difference may be attributed to the high efficiency of SARS-CoV-2 S activation and its extensive adaptation to host proteases, which could reduce neutralization efficacy. These findings provided a valuable strategy for preventing SC2r-CoVs in cross-species transmission.

## DISCUSSION

The threat to human health posed by coronaviruses primarily stems from cross-species transmission, with all seven known human-infecting coronaviruses originating from animals. SARS-CoV-2 caused an unprecedented threat to human health during its pandemic. Extensive efforts have been made worldwide to trace the zoonotic origin of SARS-CoV-2. RaTG13, isolated from *R. affinis,* was the first virus reported to be closely related to SARS-CoV-2, sharing approximately 96% genomic sequence identity (37). Subsequently, numerous SC2r-CoVs have been identified in various bat species, including *R. malayanus* (30, 37), *R. marshalli* (30), *R. cornutus* (38), *R. acuminatus* (39), and *R. pusillus* (40), as well as in pangolins (41, 42). These findings not only confirm the animal origin of SARS-CoV-2 but also highlight the potential risk associated with the cross-species transmission of the many SC2r-CoVs circulating in the wild. To better understand the mechanisms underlying the cross-species transmission of SC2r-CoVs, further research is needed into the characteristics of their spike proteins and cell entry mechanisms.

BANAL coronaviruses have been identified as the closest relatives to SARS-CoV-2, particularly in terms of their spike proteins (30). The RBDs of BANAL-52 and BANAL-103 exhibit the highest sequence similarity to the SARS-CoV-2 RBD, with only one amino acid difference (Q498H) in the RBM (Fig. S1). This difference has been reported not to destabilize the interface between the BANAL RBD and hACE2 (30), which aligns with our SPR results regarding viral RBD-ACE2 interactions (Fig. 1C through 1H). Despite the very high sequence identity among the RBDs of SARS-CoV-2, BANAL-52, and BANAL-103, their S pseudovirions have been shown to display varying levels of cell entry efficiency when using ACE2 from different bat species (34). In this study, we observed significant variation in the spike-ACE2 interaction for SARS-CoV-2, BANAL-52, and BANAL-103 (Fig. 1I through 1P), which may explain differences in S-mediated cell entry and could be associated with the capacity for conformational changes in spike proteins.

Structural analysis revealed that LA binding stabilizes the SARS-CoV-2 RBD conformational change, preventing spike-mediated cell entry (15). LA binding was also observed in BANAL-52 and a SARS-related bat coronavirus BM48-31 spike but was not detected in BANAL-236 or other SARS-related bat coronavirus spikes, according to a recent structural study (43). Additionally, LA binding has not been reported in other previously studied SARS-CoV-2-related coronavirus spikes (7, 44). The reasons for differences in LA binding to Sarbecovirus spikes remain unclear, despite the conservation of LA binding pockets among these spikes.

Our study demonstrated that the BANAL-52 S, but not BANAL-103 S, binds with LA in a locked conformation (Fig. 3 and 4). Furthermore, the previously reported low pH-treated BANAL-52 spike structure showed no LA binding (34) (Fig. S5B), suggesting that low pH treatment or Golgi low pH conditions may trigger the release of LA, enabling a conformational transition of the spike from “locked” to “closed,” thereby facilitating subsequent conformational changes and receptor recognition. Based on the structural analysis, we identified that the side chain conformation of Y365 might be associated with LA movement in or out of its pocket (Fig. 4 and Fig. S5). Y365 has also been reported as a key residue forming the gating helix of the LA pocket in the SARS-CoV-2 spike (30). Besides LA binding, additional N-linked glycosylation in the RBD region has been shown to influence the conformational dynamics of the SARS-CoV-2 spike (7). Compared to BANAL-52 and other SARS-CoV-2-related coronavirus spikes, the BANAL-103 spike contains two extra N-glycans linked to its RBD conformational change (Fig. 4), which stabilize the spike in a closed conformation.

It has been shown that BANAL coronavirus spikes can utilize a wide range of animal ACE2 receptors and mediate pseudovirus entry *in vitro* (34, 45). And, we found that ACE2 with K31 enhances the binding of the BANAL-52 spike (Fig. S2). However, we observed that the cell-cell fusion mediated by the spikes of BANAL-52 and BANAL-103 is not as efficient as that of the SARS-CoV-2 spike (Fig. 5), which is attributed to their relatively limited spectrum of host protease utilization (Fig. 5 and Fig. S7). Although protease usage varies across different cells, it still represents a narrower range compared to SARS-CoV-2 (34, 35). The acquisition of a furin cleavage site in BANAL spikes significantly increases viral entry. Proteolysis activates the spike and promotes cell-cell fusion, potentially shifting the tropism of BANAL-236 toward intestinal cells rather than respiratory cells (46).

The spike RBD is a key antigen for neutralizing antibodies (36). Previous studies in mice have confirmed the cross-immunogenicity between SARS-CoV-2 and BANAL S (34). In this study, we evaluated the neutralizing antibody levels in sera collected during health check-ups from individuals who were either vaccinated against SARS-CoV-2 or had recovered from infection. This assessment provides insights into the population’s immune status against BANAL viruses. Our results indicate that these sera exhibit robust cross-neutralization against BANAL viruses, yet the antibody titers remain relatively low in the sera (Fig. 6). These findings suggest that SARS-CoV-2-related vaccines may offer protection against BANAL viruses. However, neutralizing antibody levels have significantly declined in the general population, raising concerns about potential cross-species transmission events.

**Fig 7:**
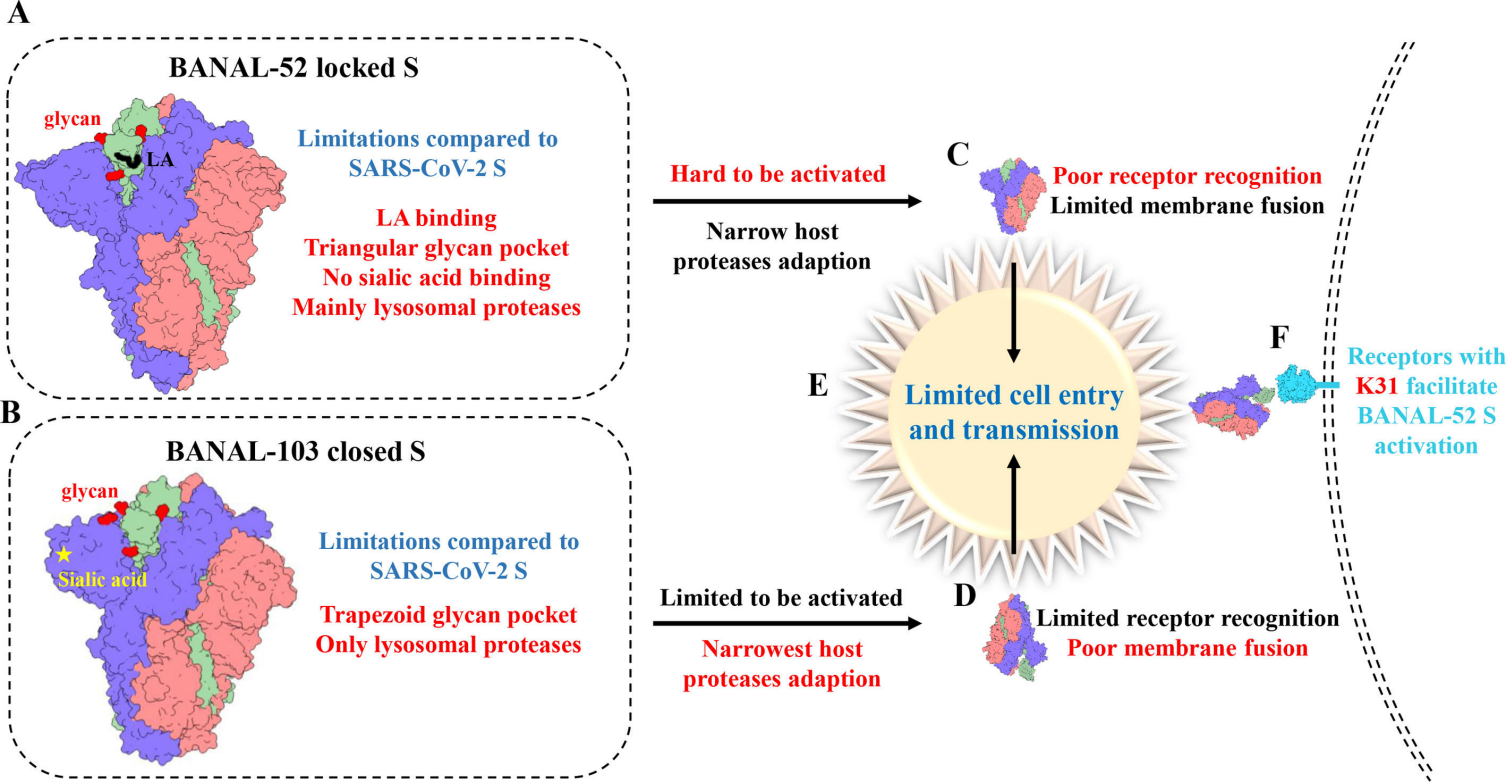
Summary of limitations regarding BANAL spike-mediated cell entry and cross-species transmission.

In conclusion, the two SARS-CoV-2 closest bar coronaviruses (BANAL-52 and BANAL-103) were systematically investigated in terms of spike structures, restriction of S activation, host protease adaptation, and cross-antigenicity. This analysis clarified the factors that limit BANAL viruses’ cell entry and cross-species transmission (Fig. 7). This study provides insights into BANAL virus spike-receptor interaction, cell entry mechanisms, and the potential for cross-species transmission.

## MATERIALS AND METHODS

### Cell lines and plasmids

HEK293T, HeLa, Calu-3, and MRC-5 cells were obtained from the American Type Culture Collection (ATCC) and cultured in Dulbecco’s modified Eagle medium supplemented with 10% fetal bovine serum, 2 mM L-glutamine, 100 units/mL penicillin, and 100 µg/mL streptomycin (Life Technologies). The HEK293 T-hACE2 cell line stably expresses human ACE2 and was derived from HEK293T cells. The HEK293F cell line was obtained from Union-Biotech and cultured under shaking conditions in Union293 medium with 8% CO2.

The full-length SARS-CoV-2 spike (GenBank accession number QHD43416.1), BANAL-52 Spike (GenBank accession number UAY13217.1), BANAL-103 spike (GenBank accession number UAY13229.1), human ACE2 (GenBank accession number NM_021804), and *Rhinolophus pusillus* bat ACE2 (GenBank accession number ADN93477.1) were synthesized (Sangon Biotech) and cloned into the pcDNA3.1(+) vector (Life Technologies) with a C-terminal C9 tag.

The ectodomains of BANAL-52 spike (residues 14–1,207 with two proline mutations, K982P and V982P) and BANAL-103 spike (residues 14–1,206 with two proline mutations, K978P and V979P) were subcloned into pcDNA3.1(+) vector, incorporating an N-terminal CD5 signal peptide, a C-terminal foldon trimerization peptide, and a His_8_ tag.

The following fragments were subcloned into the pcDNA3.1(+) vector with an N-terminal CD5 signal peptide and a C-terminal His_8_ tag, including SARS-CoV-2 S-NTD (residues 14 to 316), BANAL-52 S-NTD (residues 14 to 316), BANAL-103 S-NTD (residues 14 to 312), SARS-CoV-2 S-RBD (residues 330 to 530), BANAL-52 S-RBD (residues 330 to 530), BANAL-103 S-RBD (residues 326 to 526), human ACE2 ectodomain (residues 18 to 615), and *Rhinolophus pusillus* bat ACE2 ectodomain (residues 18 to 615).

### Protein expression and purification

All proteins were produced in HEK293F cells via transient transfection. The protein was harvested from the cell culture medium 4–5 days post-transfection and subsequently purified using an Ni-NTA column followed by a Superdex200 gel filtration column (GE Healthcare), as described previously (47). The purified proteins were stored in a buffer containing 20 mM Tris (pH 7.2) and 200 mM NaCl for subsequent use.

### Surface plasmon resonance assay

SPR assays were performed using a Biacore 1K system (Cytiva), as previously described 49 (48). Briefly, hACE2 or Rp-bACE2 was covalently immobilized to a CM5 sensor chip via their amine groups (Cytiva). The running buffer consisted of 10 mM HEPES pH 7.4, 150 mM NaCl, 3 mM EDTA, and 0.05% Tween-20. Purified recombinant S RBD was serially diluted and injected at concentrations ranging from 5 to 85 nM. For analysis of the binding affinity of S1-NTD to bovine mucin, each S1-NTD was covalently immobilized to a CM5 sensor chip through its amine groups (Cytiva). Serial dilutions of bovine mucin were injected at concentrations ranging from 2.3 to 300 nM. The resulting data were fitted to a 1:1 binding model using Biacore Evaluation Software (Cytiva).

### Protein pull-down assay

A protein pull-down assay was conducted using the Dynabeads immunoprecipitation kit (Thermo Fisher Scientific), as previously described (48). Briefly, 50 µL of Dynabeads was washed with phosphate-buffered saline (PBS) buffer and then incubated with 5 µg of His-tagged protein on a roller at room temperature for 30 min. Subsequently, the protein-bound beads were washed three times with 1 mL of PBS buffer plus 0.05% Tween-20 (PBST) on a roller for 10 min each and then were aliquoted into separate tubes for further use. To prepare cell-associated coronavirus spike or ACE2 protein, HEK293T cells were transfected with pcDNA3.1(+) plasmid encoding either the coronavirus spike or ACE2 (each containing a C-terminal C9 tag); 48 h post-transfection, the spike-expressing or ACE2-expressing cells were lysed using a sonicator in lysis buffer and centrifuged at 12,000 × *g* for 2 min. The supernatants were incubated with the relevant protein-bound beads in 2 mL tubes on a roller at room temperature for 1 h. Then beads were washed three times with PBST buffer, and the bound proteins were eluted using an elution buffer. Finally, the samples were analyzed by Western blot and detected using an anti-C9 tag antibody or anti-His tag antibody.

### Proteolysis assay

The proteolysis assay was performed as previously described (18). The specified pseudoviruses were purified using a 10%–30% sucrose gradient ultracentrifugation at 250,000 × *g* at 4°C for 2 hours. The purified pseudoviruses were incubated either alone or with an excess of recombinant hACE2 at 37°C for 30 min. Following this, the indicated pseudoviruses were treated with 1ÍÍ10-3^−3^ mg/mL trypsin on ice for 20 min. The reaction was terminated by adding an excess of soybean trypsin inhibitor. Samples were then applied for Western blot analysis using an antibody against the C-terminal C9 tag of the respective spikes.

### Flow cytometry assay

The recombinant SARS-CoV-2/BANAL-52/BANAL-103 RBD or S protein was evaluated for its cell-binding capacity using flow cytometry, as previously described (47). Briefly, HEK293T cells expressing ACE2 were incubated with the indicated recombinant coronavirus RBD or S protein, each containing a C-terminal His_8_ tag (at a concentration of 50 µg/mL), at 4 °C on a roller for 1 h. The samples were then washed three times with pre-cooled PBS and subsequently incubated with TITC-conjugated anti-His antibody for 1 h. Finally, the cells were analyzed by flow cytometry to assess the binding of the RBD or S protein.

### Coronavirus-spike-mediated pseudovirus entry assay

Retroviruses pseudotyped with coronavirus spike were generated as previously described (29). Briefly, the pseudoviruses were produced by co-transfecting HEK293T cells with a plasmid carrying an Env-defective, luciferase-expressing HIV-1 genome (pNL4-3.luc.RE) and a plasmid encoding the coronavirus spike. The pseudoviruses were harvested 72 hours post-transfection. The pseudoviruses were subsequently used to infect the indicated cells. After incubation at 37°C for 5 hours, the medium was replaced, and the cells were further incubated for 60 hours. The cells were then washed with PBS and lysed. Aliquots of cell lysates were transferred to Optiplate-96 (Tecan Group Ltd.), followed by the addition of the luciferase substrate. Relative light units (RLUs) were measured using a multifunction plate reader (Tecan Group Ltd.). All the measurements were carried out in triplicate.

Inhibition of pseudovirus entry using various protease inhibitors was performed as previously described (31). In brief, target cells were pre-incubated with the medium containing a final concentration of 30 µM camostat (Sigma-Aldrich), an inhibitor of TMPRSS2, 30 µM E64d (Sigma-Aldrich), an inhibitor of lysosomal cathepsins, 30 µM PPC (proprotein convertase) inhibitor (Sigma-Aldrich), 30 µM trypsin inhibitor (Sigma-Aldrich), or DMSO (negative control) at 37°C for 1 h. The cells were then infected with pseudoviruses and incubated at 37°C for 6 to 8 h before the medium was replaced with fresh DMEM. After an additional 48 h, the cells were lysed, and luciferase activity was measured.

### Cryo-electron microscopy sample preparation and data acquisition

For sample preparation, aliquots of BANAL-52 or BANAL-103 S-e (3 µL, 0.35 mg/mL, in a buffer containing 2 mM Tris, pH 7.2, and 20 mM NaCl) were applied to glow-discharged C-flat Au grids (CF-1.2/1.3 300 mesh, Protochips). The grids were blotted for 2–4 s (blot force 1) and then plunge-frozen in liquid ethane using n FEI Mark IV Vitrobot system (FEI Company).

For data collection, images were recorded using a Falcon 4i direct electron detector (Thermo Fisher Scientific) mounted to a 300 kV Titan-Krios G3i transmission electron microscope (Thermo Fisher Scientific). Data acquisition was performed using EPU software at ×96,000 magnification, yielding a pixel size of 0.83 Å and a defocus range from 0.5 to 2.5 µM. Each movie had a total accumulated exposure of 50 e/Å2^2^ fractionated in 40 frames with an exposure time of 4.97 s. Data collection statistics are summarized in Table S1.

### Cryo-electron microscopy data processing

Schematic workflows for data processing are presented in Fig. S7. A total of 4,776 movies were collected for BANAL-52 Se, and 4,331 movies were collected for BANAL-103 Se. Beam-induced motion correction and dose weighting were performed using MotionCor2 (49). For the BANAL-52 Se data set, the final image was binned to a pixel size of 1.66, yielding a higher resolution for the final refined map. The parameters of the microscope contrast transfer function were estimated for each micrograph using Gctf51 (50). All particles were automatically picked using templates generated from approximately 500 manually picked particles in RELION 4.0 on a GPU workstation.

For BANAL-52 Se, 364,305 particles were auto-picked and extracted. An initial round of 2D classification with 100 classes was performed in RELION 4.0, but no clear target classes were isolated. Subsequently, all particles were subjected to 3D classification (C1) using a template generated by a 3D initial model in RELION 4.0 based on the original manually picked particles. One class containing 257,412 particles was selected for the next round of 3D classification (C1). From this, one class with 39,969 particles was chosen and projected into 2D classification. The best classes, comprising 28,491 particles, were then selected for another round of 3D classification (C3). Two classes with 20,175 particles were chosen for the final 3D refinement (C3), yielding a 3.51 Å cryo-EM map.

For BANAL-103 Se, 340,844 particles were auto-picked, extracted, and subjected to a round of 2D classification with 100 classes in RELION 4.0. From this, 96,956 particles from the best classes were selected and projected into a round of 3D classification (C1). Two out of three classes, containing 81,886 particles, were selected for another round of 3D classification (C3). Finally, 52,916 particles from the best classes were selected and used for 3D refinement (C3), resulting in the final 3.11 Å cryo-EM map.

All the maps were sharpened with a solvent mask using RELION 4.0 postprocessing. The reported resolution was based on the gold-standard Fourier shell correlation (FSC) of 0.143, and Fourier shell correction curves were corrected for the effects of soft masking using high-resolution noise substitution (51). Data processing statistics are summarized in Table S1.

### Model building and refinement

BANAL-52 and BANAL-103 Se models were predicted by SWISS-MODEL using a template of SARS-CoV-2 spike (PDB ID: 6ZB5). The predicted models were then docked into the cryo-EM map using UCSF Chimera (52). Manual model rebuilding was performed in Coot using the tool of Real-Space Refinement Zone, guided by the well-defined continuous density of the main chain (53). Side-chain assignments were guided through the densities of N-linked glycans and bulky amino acid residues. Coordinates were then refined using Phenix real_space_refinement with geometry restraints and three-fold noncrystallographic symmetry constraints (54). Refinement and model rebuilding were carried out iteratively until no further improvements were achieved in geometry parameters and model-map correlation coefficient. The final model quality was assessed using MolProbity (55), and the validation statistics of the structural models are summarized in Table S1.

### Serum cross-neutralization assay

Plasma samples were collected from participants during medical examination at the clinical laboratory as part of an ongoing study at Children’s Hospital of Chongqing Medical University, China. Participants were enrolled after receiving informed consent in their primary language. Limited clinical information, including demographic data, was collected from the participants.

The ELISA assay against the spike protein was used to evaluate the antibody titer, as previously described (56). Briefly, the indicated spike protein RBD or bovine serum albumin (BSA) was coated onto high-binding 96-well ELISA plates at a concentration of 1 ug/mL and incubated overnight at 4°C. The plates were then blocked with 3% non-fat milk for 1 h at 37  °C, followed by the addition of diluted serum samples (1:100 dilution) and further incubation for 1 h at 37  °C. After washing, the plates were incubated with horseradish-peroxidase-conjugated anti-human-IgG-Fab polyclonal antibody (1:5,000 dilution) (Jackson Immunology Lab). The enzymatic reaction was carried out using substrate 3,3′,5,5′-tetramethylbenzidine (Yeasen Inc.) and stopped with 1 N H2_2_SO4_4_. Absorbance at 450  nm (A_450_) was measured using an ELISA Plate Reader (Tecan Group Ltd.).

For the cross-neutralization assay, the pseudovirus was pre-incubated with diluted serum and then applied for the pseudovirus entry assay according to the description in the pseudovirus entry assay.

### Statistical analysis

All experiments were repeated at least three times. Statistical analyses were conducted using *t* tests. A *P* value < 0.05 was considered statistically significant, with ***P < 0.001, **P < 0.01, *P < 0.05.

## Data Availability

All relevant data are included in the article and its supplemental material. The cryo-EM maps of BANAL-52 and BANAL-103 have been deposited in the Electron Microscopy Data Bank (EMD) under accession codes EMD-62143 and EMD-62145, respectively. Additionally, the atomic models of BANAL-52 and BANAL-103 have been deposited in the Protein Data Bank (PDB) under accession codes 9K6Z and 9K75, respectively.

## SUPPLEMENTAL MATERIAL

Supplemental material jvi.01007-25-s0001.pdf Figures S1 to S8; Table S1.

ASM does not own the copyrights to Supplemental Material that may be linked to, or accessed through, an article. The authors have granted ASM a non-exclusive, world-wide license to publish the Supplemental Material files. Please contact the corresponding author directly for reuse.
