# Supplementary material for: Structural and functional constraints on spike activation and host protease utilization limit cell entry of SARS-CoV-2-related bat coronaviruses
Source: J Virol. 2025 Jul 24;99(8):e01007-25. doi: 10.1128/jvi.01007-25 (PMC12363188; doi:10.1128/jvi.01007-25)
Supplement: Supplemental material — Figures S1 to S8; Table S1. [file jvi.01007-25-s0001.pdf]

Figure S1

A

Homology (%)	SARS-CoV-2				
	Spike	S1-NTD	RBD	RBM	S2
BANAL-52	98.43	98.11	97.67	98.59	98.79
BANAL-103	90.66	67.40	97.16	98.59	98.79
BANAL-236	90.66	67.40	96.68	97.18	98.93
RaTG13	97.41	98.74	89.57	76.06	99.06
GX_P5L	92.38	88.05	87.14	76.06	95.83
RpYN06	81.31	65.09	66.67	38.60	91.67
RacCS203	72.75	41.61	63.81	36.84	88.84

B

```
SARS-CoV-2    333 TNLCPFG EVFNATRFAS VYAWNRKRIS NCVADYSVLY 369
BANAL-52      333 TNLCPFG EVFNATTFAS VYAWNRKRIS NCVADYSVLY 369
BANAL-103     329 TNLCPFG EVFNATTFAS VYAWNRKRIS NCVADYSVLY 365
                  ******* ****** *** ********** **********

SARS-CoV-2      NSASFSTFKC YGVSPTKLND LCFTNVYADS FVIRGDEVRQ 409
BANAL-52        NSTSFSTFKC YGVSPTKLND LCFTNVYADS FVVRGDEVRQ 409
BANAL-103       NSTSFSTFKC YGVSPTKLND LCFTNVYADS FVVRGDEVRQ 405
                **:******* ********** ********** **:*******

SARS-CoV-2      IAPGQTGKIA DYNYKLPDDF TGCVIAWNSN NLDSKVGGNY 449
BANAL-52        IAPGQTGKIA DYNYKLPDDF TGCVIAWNSN NLDSKVGGNY 449
BANAL-103       IAPGQTGKIA DYNYKLPDDF TGCVIAWNSN NLDSKVGGNY 445
                ********** ********** ********** **********

SARS-CoV-2      NYLYRLFRKS NLKPFERDIS TEIYQAGSTP CNGVEGFNCY 489
BANAL-52        NYLYRLFRKS NLKPFERDIS TEIYQAGSTP CNGVEGFNCY 489
BANAL-103       NYLYRLFRKS NLKPFERDIS TEIYQAGSTP CNGVEGFNCY 485
                ********** ********** ********** **********

SARS-CoV-2      FPLQSYGFQP TNGVGYQPYR VVVLSFELLH APATVCGPKK 529
BANAL-52        FPLQSYGFHP TNGVGYQPYR VVVLSFELLN APATVCGPKK 529
BANAL-103       FPLQSYGFHP TNGVGYQPYR VVVLSFELLN APATVCGPKK 525
                ********:* ********** *********: **********
```

C

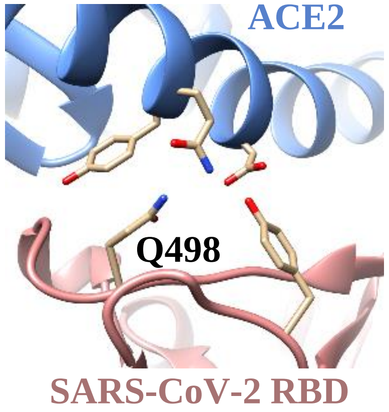

D

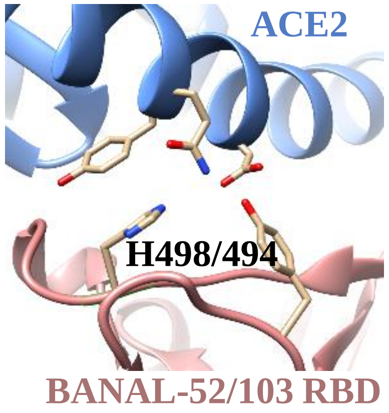

Figure S2

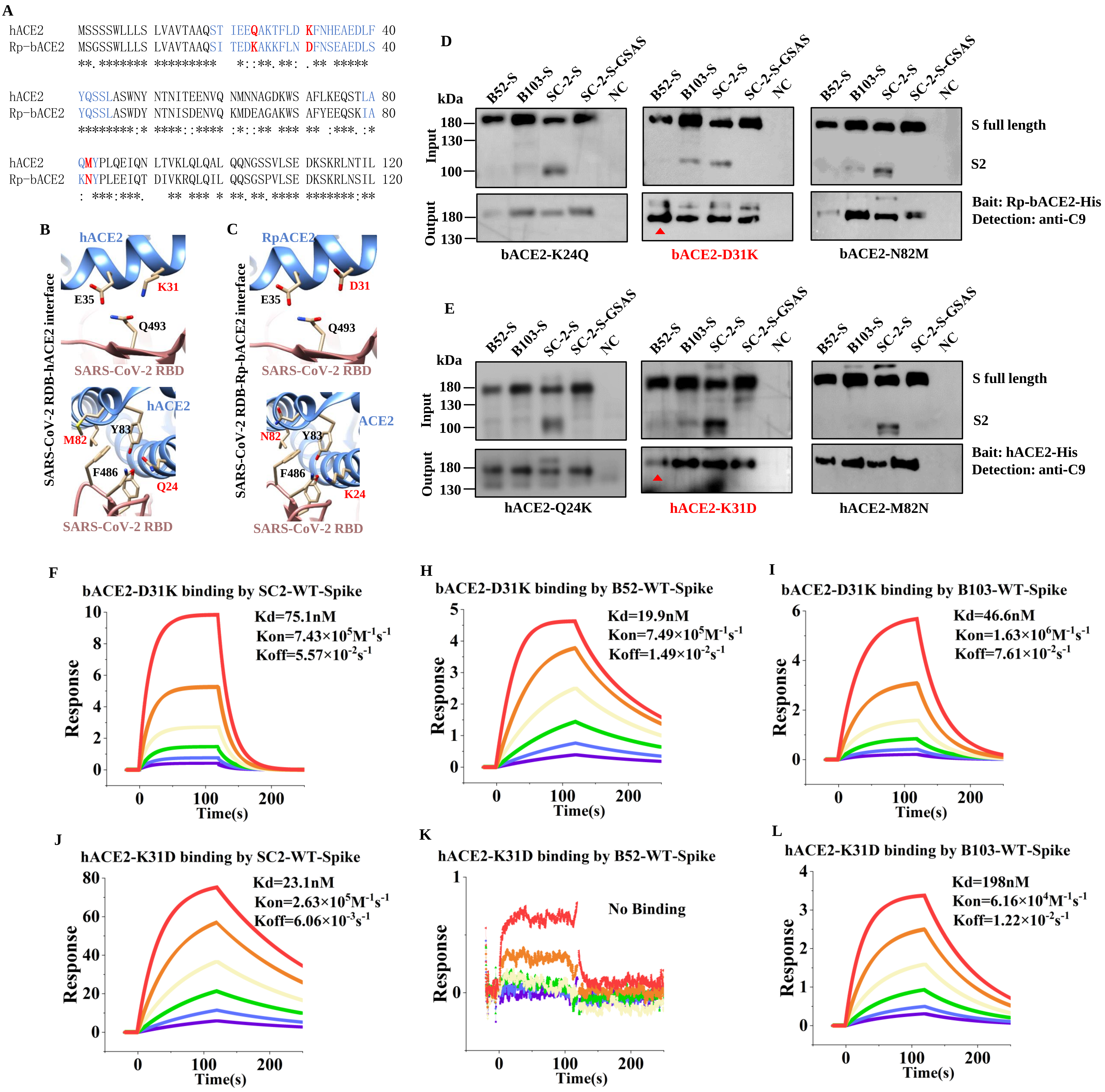
A
hACE2 MSSSSWLLLS LVAVTAAQST IEEQAKTFLD KFNHEAEDLF 40
Rp-bACE2 MSGSSWLLLS LVAVTAAQSI TEDKAKKFLN DFNSEAEDLS 40
hACE2 YQSSLASWNY NTNITEENVQ NMNNAGDKWS AFLKEQSTLA 80
Rp-bACE2 YQSSLASWDY NTNISDENVQ KMDEAGAKWS AFYEEQSKIA 80
hACE2 QMYPLQEIQN LTVKLQLQAL QQNGSSVLSE DKSKRLNTIL 120
Rp-bACE2 KNYPLEEIQT DIVKRQLQIL QQSGSPVLSE DKSKRLNSIL 120
B
hACE2
K31
E35
Q493
SARS-CoV-2 RBD
M82
Y83
F486
Q24
SARS-CoV-2 RDB-hACE2 interface
C
RpACE2
D31
E35
Q493
SARS-CoV-2 RBD
ACE2
N82
Y83
F486
K24
SARS-CoV-2 RDB-Rp-bACE2 interface
D
B52-S
B103-S
SC-2-S
SC-2-S-GSAS
NC
kDa
180
130
100
Input
Output
bACE2-K24Q
bACE2-D31K
bACE2-N82M
S full length
S2
Bait: Rp-bACE2-His
Detection: anti-C9
E
hACE2-Q24K
hACE2-K31D
hACE2-M82N
Bait: hACE2-His
Detection: anti-C9
F
bACE2-D31K binding by SC2-WT-Spike
Kd=75.1nM
Kon=7.43×10^5M^-1s^-1
Koff=5.57×10^-2s^-1
H
bACE2-D31K binding by B52-WT-Spike
Kd=19.9nM
Kon=7.49×10^5M^-1s^-1
Koff=1.49×10^-2s^-1
I
bACE2-D31K binding by B103-WT-Spike
Kd=46.6nM
Kon=1.63×10^6M^-1s^-1
Koff=7.61×10^-2s^-1
J
hACE2-K31D binding by SC2-WT-Spike
Kd=23.1nM
Kon=2.63×10^5M^-1s^-1
Koff=6.06×10^-3s^-1
K
hACE2-K31D binding by B52-WT-Spike
No Binding
L
hACE2-K31D binding by B103-WT-Spike
Kd=198nM
Kon=6.16×10^4M^-1s^-1
Koff=1.22×10^-2s^-1
Response
Time(s)

Figure S3

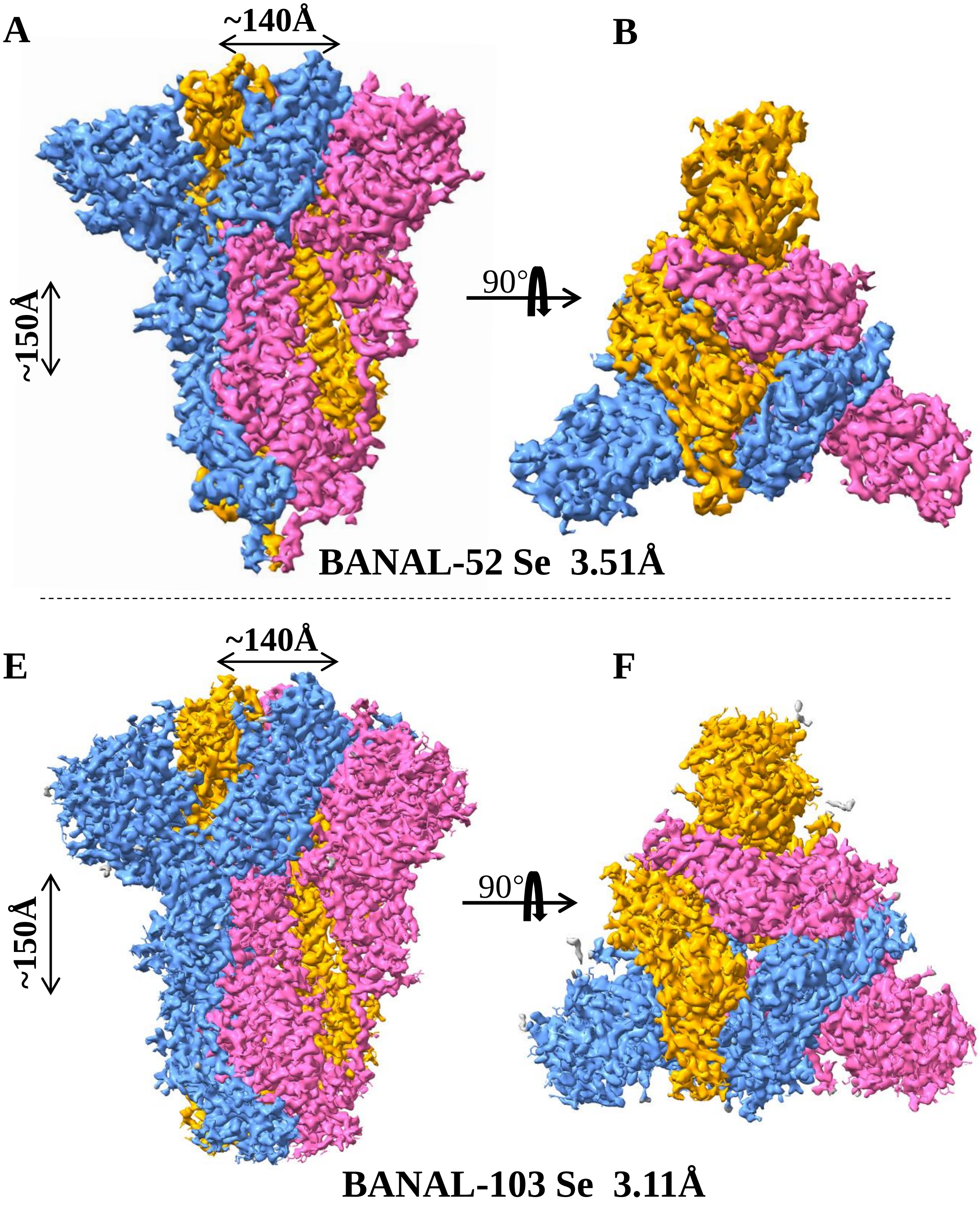

A
~140Å
~150Å
B
90°
BANAL-52 Se 3.51Å
E
~140Å
~150Å
F
90°
BANAL-103 Se 3.11Å

Figure S4

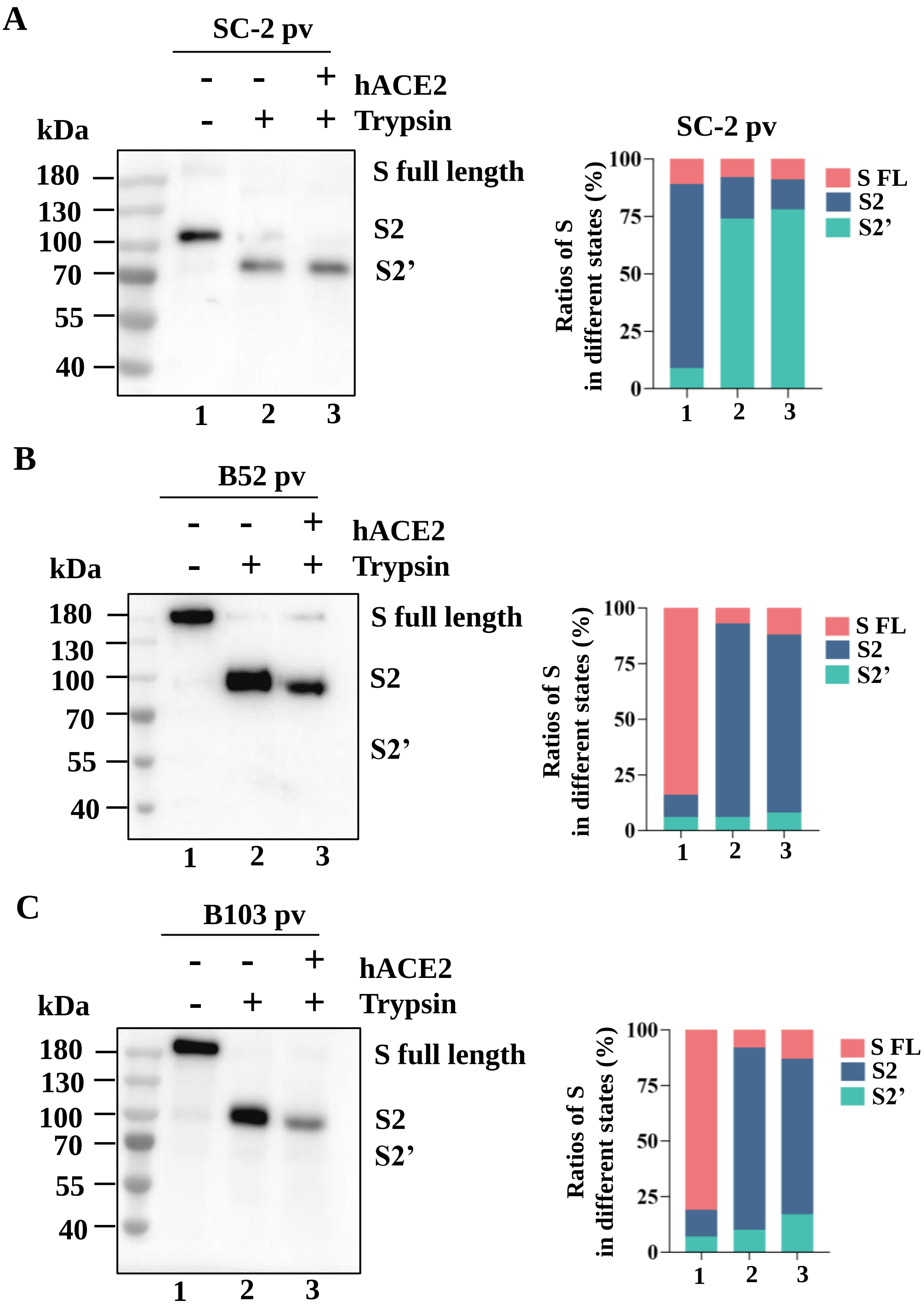
A
SC-2 pv
- - + hACE2
- + + Trypsin
kDa
180
130
100
70
55
40
S full length
S2
S2’
1 2 3
SC-2 pv
Ratios of S in different states (%)
100
75
50
25
0
1 2 3
S FL
S2
S2’
B
B52 pv
- - + hACE2
- + + Trypsin
kDa
180
130
100
70
55
40
S full length
S2
S2’
1 2 3
Ratios of S in different states (%)
100
75
50
25
0
1 2 3
S FL
S2
S2’
C
B103 pv
- - + hACE2
- + + Trypsin
kDa
180
130
100
70
55
40
S full length
S2
S2’
1 2 3
Ratios of S in different states (%)
100
75
50
25
0
1 2 3
S FL
S2
S2’

Figure S5

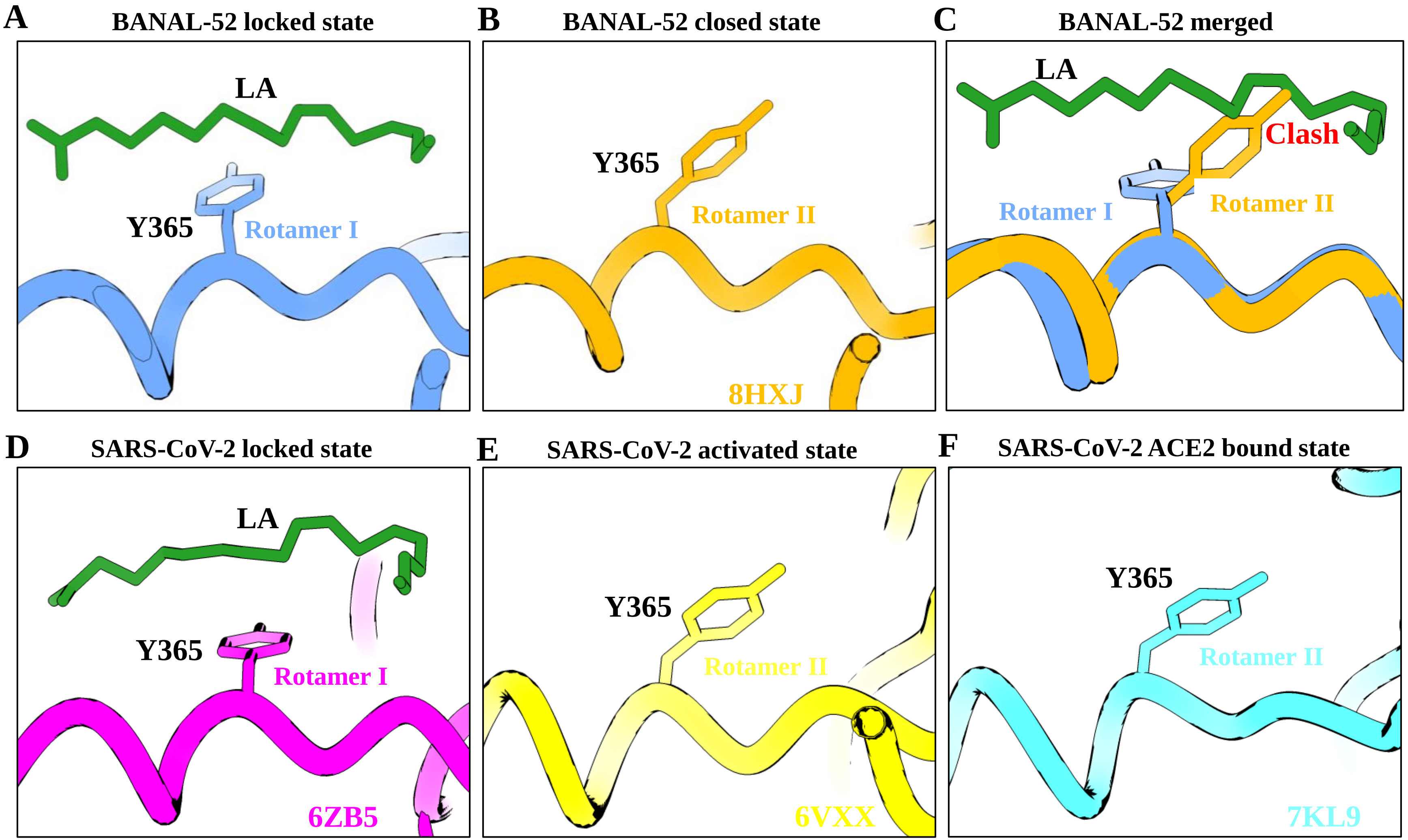
A
BANAL-52 locked state
LA
Y365
Rotamer I
B
BANAL-52 closed state
Y365
Rotamer II
8HXJ
C
BANAL-52 merged
LA
Clash
Rotamer I
Rotamer II
D
SARS-CoV-2 locked state
LA
Y365
Rotamer I
6ZB5
E
SARS-CoV-2 activated state
Y365
Rotamer II
6VXX
F
SARS-CoV-2 ACE2 bound state
Y365
Rotamer II
7KL9

Figure S6

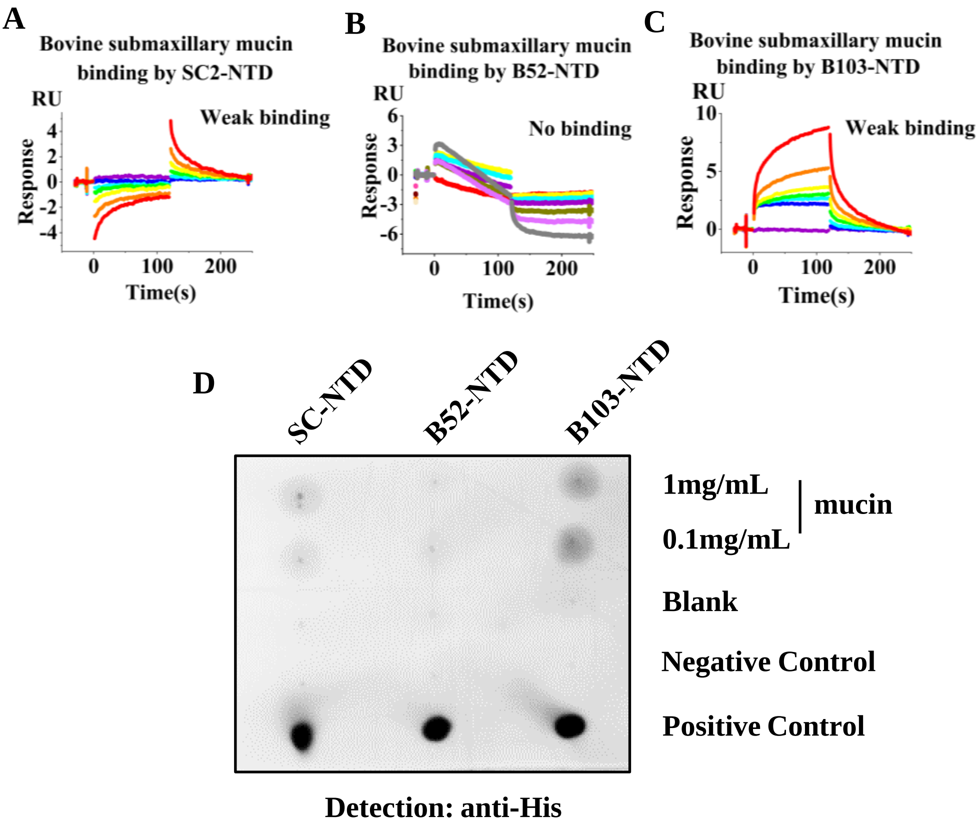
A
Bovine submaxillary mucin binding by SC2-NTD
RU
Weak binding
Response
4
2
0
-2
-4
0
100
200
Time(s)
B
Bovine submaxillary mucin binding by B52-NTD
RU
6
3
0
-3
-6
No binding
Response
0
100
200
Time(s)
C
Bovine submaxillary mucin binding by B103-NTD
RU
10
5
0
Weak binding
Response
0
100
200
Time(s)
D
SC-NTD
B52-NTD
B103-NTD
1mg/mL
0.1mg/mL
mucin
Blank
Negative Control
Positive Control
Detection: anti-His

Figure S7

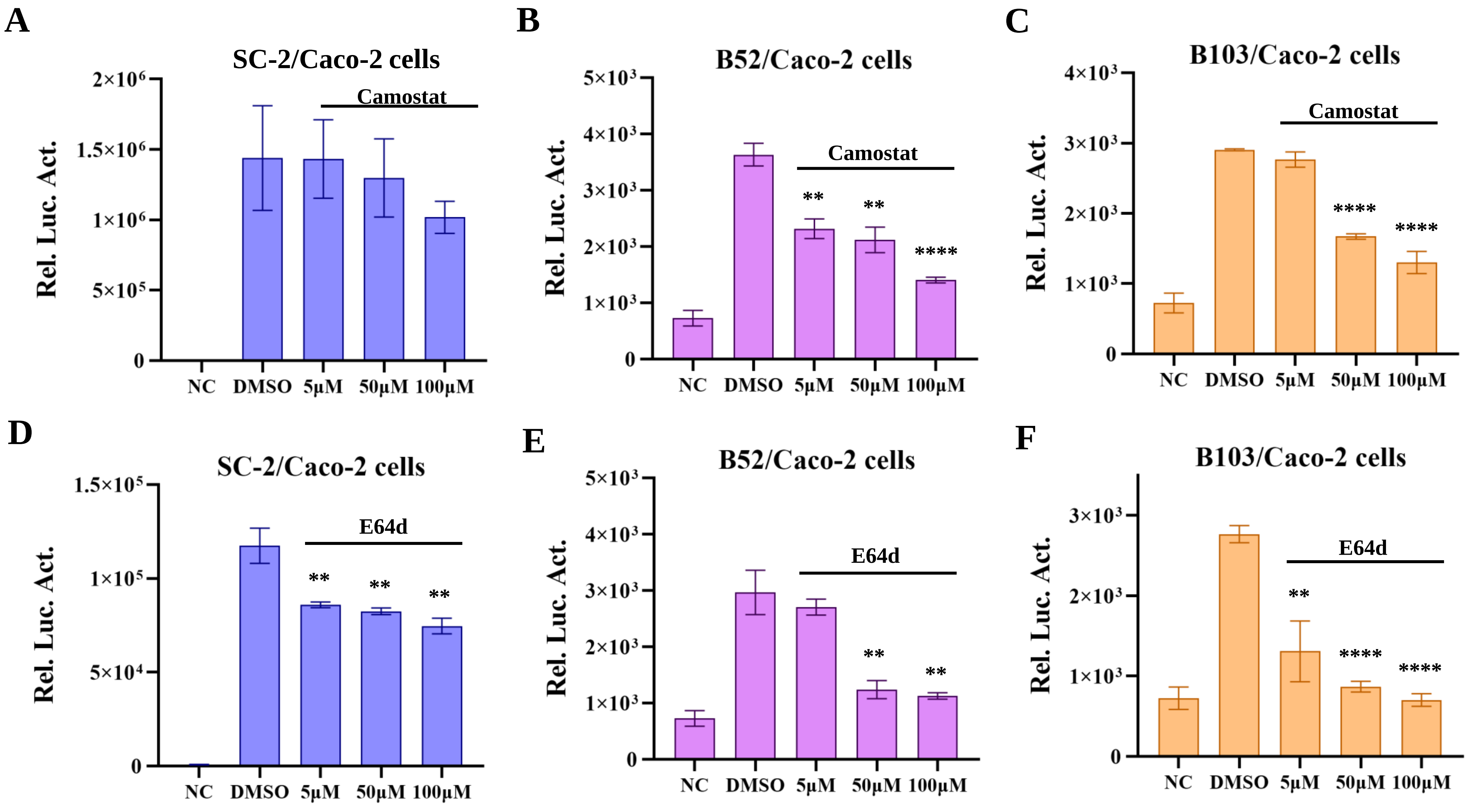
A
SC-2/Caco-2 cells
Camostat
Rel. Luc. Act.
2×10⁶
1.5×10⁶
1×10⁶
5×10⁵
0
NC DMSO 5μM 50μM 100μM
B
B52/Caco-2 cells
Camostat
Rel. Luc. Act.
5×10³
4×10³
3×10³
2×10³
1×10³
0
**
**
****
NC DMSO 5μM 50μM 100μM
C
B103/Caco-2 cells
Camostat
Rel. Luc. Act.
4×10³
3×10³
2×10³
1×10³
0
****
****
NC DMSO 5μM 50μM 100μM
D
SC-2/Caco-2 cells
E64d
Rel. Luc. Act.
1.5×10⁵
1×10⁵
5×10⁴
0
**
**
**
NC DMSO 5μM 50μM 100μM
E
B52/Caco-2 cells
E64d
Rel. Luc. Act.
5×10³
4×10³
3×10³
2×10³
1×10³
0
**
**
NC DMSO 5μM 50μM 100μM
F
B103/Caco-2 cells
E64d
Rel. Luc. Act.
3×10³
2×10³
1×10³
0
**
****
****
NC DMSO 5μM 50μM 100μM

Figure S8

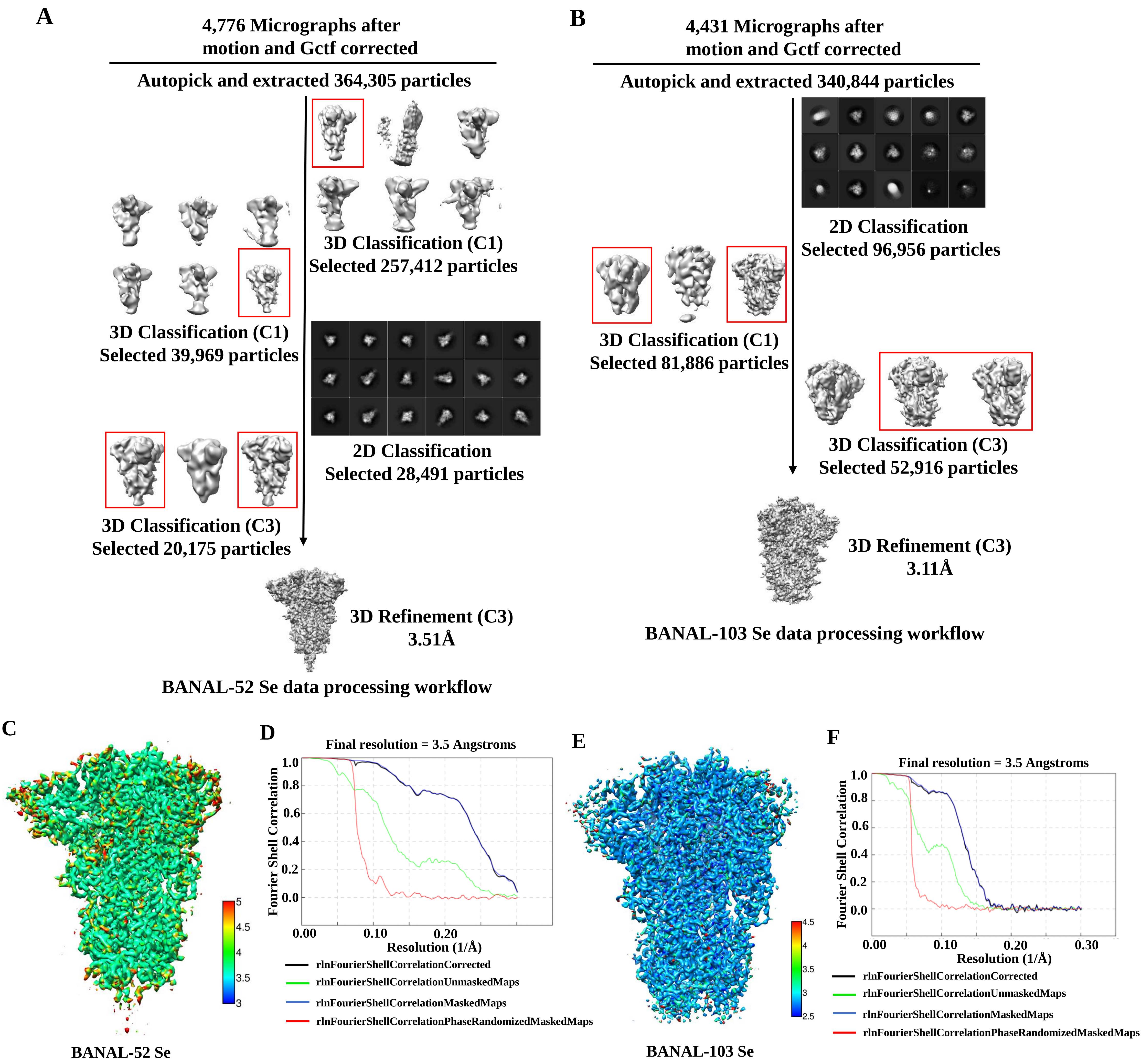
A
4,776 Micrographs after
motion and Gctf corrected
Autopick and extracted 364,305 particles
3D Classification (C1)
Selected 257,412 particles
3D Classification (C1)
Selected 39,969 particles
2D Classification
Selected 28,491 particles
3D Classification (C3)
Selected 20,175 particles
3D Refinement (C3)
3.51Å
BANAL-52 Se data processing workflow
B
4,431 Micrographs after
motion and Gctf corrected
Autopick and extracted 340,844 particles
2D Classification
Selected 96,956 particles
3D Classification (C1)
Selected 81,886 particles
3D Classification (C3)
Selected 52,916 particles
3D Refinement (C3)
3.11Å
BANAL-103 Se data processing workflow
C
5
4.5
4
3.5
3
BANAL-52 Se
D
Final resolution = 3.5 Angstroms
Fourier Shell Correlation
1.0
0.8
0.6
0.4
0.2
0.0
0.00
0.10
0.20
Resolution (1/Å)
rlnFourierShellCorrelationCorrected
rlnFourierShellCorrelationUnmaskedMaps
rlnFourierShellCorrelationMaskedMaps
rlnFourierShellCorrelationPhaseRandomizedMaskedMaps
E
4.5
4
3.5
3
2.5
BANAL-103 Se
F
Final resolution = 3.5 Angstroms
Fourier Shell Correlation
1.0
0.8
0.6
0.4
0.2
0.0
0.00
0.10
0.20
0.30
Resolution (1/Å)
rlnFourierShellCorrelationCorrected
rlnFourierShellCorrelationUnmaskedMaps
rlnFourierShellCorrelationMaskedMaps
rlnFourierShellCorrelationPhaseRandomizedMaskedMaps

Table S1: Cryo-EM data collection, refinement and validation statistics.

	BANAL-52 Se	BANAL-103 Se
**Data collection**		
Magnification	96,000	96,000
Voltage (kV)	300	300
Electron exposure (e–/Å^2)	50.00	50.00
Frames per movie	40	40
Defocus range (μm)	0.5–2.5	0.5–2.5
Pixel size (Å)	0.83	0.83
**Reconstruction**		
Symmetry imposed	C3	C3
Initial particle images (no.)	364,305	340,844
Final particle images (no.)	20,175	52,916
Map resolution (Å)	3.51	3.11
FSC threshold	0.143	0.143
Map sharpening B-factor (Å)	-76.69	-95.84
**Model Validation**		
R.m.s. deviations		
Bond lengths (Å)	0.002	0.002
Bond angles (°)	0.474	0.507
Validation		
MolProbity score	1.67	1.66
Clashscore	5.44	5.83
Poor rotamers (%)	0.07	0.42
Ramachandran plot		
Favored (%)	94.48	95.13
Allowed (%)	5.52	4.78
Outliers (%)	0.00	0.09

Supplemental Figure Legends

Figure S1. Similarities between SARS-CoV-2 and BANAL Spikes. (A) Homology between the S protein of SARS-CoV-2 and the related coronaviruses. S1-NTD: S1 N-terminal domain, RBD: receptor binding domain, RBM: receptor binding motif. (B) Sequence alignment of the RBD and RBM regions (highlighted in magenta) between SARS-CoV-2 and BANAL spikes. (C and D) The potential effects of Q498 and H498/494 on receptor binding were analyzed based on structural data (reference structure PDB:6VW1; the BANAL-52/103 RBD structure was predicted using SARS-CoV-2 RBD as a reference and docked into the 6VW1 complex structure).

Figure S2. ACE2 with K31 facilitates BANAL-52 S activation. (A) Sequence alignment of hACE2 and *Rp*-bACE2. The RBD binding region is indicated in blue, while differences in RBD-binding-associated amino acids between hACE2 and *Rp*-bACE2 are highlighted in red. (B and C) Structural analysis of the RBD-binding-associated amino acids in hACE2 and *Rp*-bACE2 (reference structure PDB:6VW1, the *Rp*-bACE2 structure was predicted using hACE2 as a reference and docked into the 6VW1 complex structure). (D) Pull-down assay analyzing the binding of BANAL-52, BANAL-103, SARS-CoV-2, and SARS-CoV-2 furin mutant S to *Rp*-bACE2 mutations. The red angle indicates a significant increase in BANAL-52 S binding. (E) Pull-down assay analyzing the binding of BANAL-52, BANAL-103, SARS-CoV-2, and SARS-CoV-2 furin mutant S to hACE2 mutations. The red angle indicates a significant

reduction in BANAL-52 S binding. (F-I) SPR assay to evaluate the binding affinity of purified recombinant *Rp*-bACE2-D31K to the S ectodomains of SARS-CoV-2 (F), BANAL-52 (H), and BANAL-103 (I). (J-L) SPR assay to evaluate the binding affinity of purified recombinant hACE2-K31D to the S ectodomains of SARS-CoV-2 (J), BANAL-52 (K), and BANAL-103 (L).

Figure S3. Cryo-EM maps of BANAL Spikes. (A and B) Side and top views of the BANAL-52 cryo-EM map at a resolution of 3.51Å. (Cand D) Side and top views of the BANAL-103 cryo-EM map at a resolution of 3.11Å.

Figure S4. Proteolysis assay of SARS-CoV-2, BANAL-52, and BANAL-103 S-pseudotyped viruses. (A-C) Western blot analysis of purified SARS-CoV-2, BANAL-52, and BANAL-103 S-pseudotyped viruses treated with trypsin in the presence or absence of hACE2, pv is the short form for S-pseudotyped viruses. The ratio of S, S2, and S2' for each is analyzed using ImageJ and presented on the right.

Figure S5. Y365 side chain conformations. (A) Y365 adopts rotamer I in the BANAL-52 locked conformation (structure resolved in this manuscript, with LA binding). (B) Y365 adopts rotamer II in the BANAL-52 closed conformation (structure previously reported, without LA binding, PDB:8HXJ). (C) Y365 in rotamer II clashes with LA when merging (A) and (B). (D-F) Y365 side chain conformations in the SARS-CoV-2 locked conformation (with LA binding, PDB:6Z5B), activated conformation

(PDB:6VXX), and ACE2-bound conformation (without LA binding, PDB:7KL9).

Figure S6. The binding of BANAL S1-NTD to mucin. (A-C) SPR analysis of the binding affinity of SARS-CoV-2, BANAL-52, and BANAL-103 S1-NTD to bovine mucin. (D) Dot blot analysis of the binding of SARS-CoV-2, BANAL-52, and BANAL-103 S1-NTD to bovine mucin.

Figure S7. Pseudovirus entry with inhibitors treatment in multiple concentrations. (A-C) Pseudoviruses of SARS-CoV-2, BANAL-52, and BANAL-103 enter Caco-2 cells treated with Camostat, a TMPRSS2 inhibitor, at concentrations of 5, 50, and 100 μM. (D-F) Pseudoviruses of SARS-CoV-2, BANAL-52, and BANAL-103 enter Caco-2 cells treated with E64d, a lysosomal cathepsin inhibitor, at concentrations of 5, 50, and 100 μM. $^{**}P<0.01$, $^{****}P<0.0001$

Figure S8. Cryo-EM 3D reconstruction details of BANAL Spikes. (A) The BANAL-52 Se data processing workflow. (B) The BANAL-103 Se data processing workflow. (C and E) The local resolution estimated by ResMap is visualized on the BANAL-52 map (C) and BANAL-103 map (E) using UCSF Chimera. (D and F) The Gold-standard resolution data for BANAL-52 (D) and BANAL-103 (F).
